# Barriers and facilitators to the implementation of digital technologies in mental health systems: a qualitative systematic review to inform a policy framework

**DOI:** 10.1186/s12913-023-10536-1

**Published:** 2024-02-26

**Authors:** Chiara Berardi, Marcello Antonini, Zephanie Jordan, Heidi Wechtler, Francesco Paolucci, Madeleine Hinwood

**Affiliations:** 1 Newcastle Business School, The University of Newcastle, Hunter St & Auckland St, 2300 Newcastle, NSW Australia; 2 School of Medicine and Public Health, The University of Newcastle, Callaghan, NSW Australia; 3 Department of Health Policy, London School of Economics and Political Science, London, WC2A 2AE UK; 4 Hunter Medical Research Institute, New Lambton Heights, NSW Australia

**Keywords:** Digital health technologies, Mental health, Health systems, Health reform

## Abstract

**Background:**

Despite the potential for improved population mental health and wellbeing, the integration of mental health digital interventions has been difficult to achieve. In this qualitative systematic review, we aimed to identify barriers and facilitators to the implementation of digital technologies in mental healthcare systems, and map these to an implementation framework to inform policy development.

**Methods:**

We searched Medline, Embase, Scopus, PsycInfo, Web of Science, and Google Scholar for primary research articles published between January 2010 and 2022. Studies were considered eligible if they reported barriers and/or facilitators to the integration of any digital mental healthcare technologies. Data were extracted using EPPI-Reviewer Web and analysed thematically via inductive and deductive cycles.

**Results:**

Of 12,525 references identified initially, 81 studies were included in the final analysis. Barriers and facilitators were grouped within an implementation (evidence-practice gap) framework across six domains, organised by four levels of mental healthcare systems. Broadly, implementation was hindered by the perception of digital technologies as impersonal tools that add additional burden of care onto both providers and patients, and change relational power asymmetries; an absence of resources; and regulatory complexities that impede access to universal coverage. Facilitators included person-cantered approaches that consider patients’ intersectional features e.g., gender, class, disability, illness severity; evidence-based training for providers; collaboration among colleagues; appropriate investment in human and financial resources; and policy reforms that tackle universal access to digital health.

**Conclusion:**

It is important to consider the complex and interrelated nature of barriers across different domains and levels of the mental health system. To facilitate the equitable, sustainable, and long-term digital transition of mental health systems, policymakers should consider a systemic approach to collaboration between public and private sectors to inform evidence-based planning and strengthen mental health systems.

**Protocol registration:**

The protocol is registered on PROSPERO, CRD42021276838.

**Supplementary Information:**

The online version contains supplementary material available at 10.1186/s12913-023-10536-1.

## Background

Although mental health disorders are associated with significantly reduced quality of life and socioeconomic burden internationally, mental healthcare systems are under-resourced and fragmented [1]. Mental health disorders affect more than 1 billion people worldwide, and make up 7% of the global burden of disease [2]. Yet, mental healthcare suffers from a major treatment gap, with more than 70% of people with mental health problems unable to access timely treatment [3]. Furthermore, multiple health, social, economic, and environmental crises tend to exacerbate socio-economic determinants of mental health [4–6]. The demand for mental healthcare regularly outstrips supply, resulting in mental health services which are crisis-driven, reactive, and over reliant on tertiary care [7].

The digitalisation of healthcare more broadly is contributing to improvements in population health and wellbeing that aligns with the third goal of the United Nations 2030 Agenda for Sustainable Development [8]. The WHO recognises both the potential for digital technologies to achieve Universal Health Coverage (UHC) [9], and the implementation challenges in both high but especially low resource settings. There is a need to develop national digital health action plans to strengthen health systems. Digital health policies showed consistent weaknesses in response to the COVID-19 pandemic [10] and require improvements in order to respond to future crises.

Digital health technologies, which include a variety of technologies that can be used either to treat patients, or to collect and share health information, have the potential to strengthen mental healthcare systems. Studies consistently show that facilitating remote consultations such as telehealth or teletherapy provides enhanced access to mental health services [11]. Electronic health records and data-driven approaches can be leveraged to enhance efficiency and integration of healthcare systems [12, 13]. The role for digital technologies in mental healthcare is increasingly being recognised and promoted by international and national initiatives, such as the WHO global strategy on digital health 2020–2025 [9], and the NICE Evidence standards framework for digital health technologies [14].

Because of its potential to reshape access to mental healthcare and improve health outcomes, digitalisation is increasingly considered to be an important determinant of health [8]. Prior systematic reviews identifying barriers and facilitators to the implementation of digital technologies in mental health care have focused on single agents’ engagement with digital technologies e.g., patients [15] or health care professionals [16]. Despite the potential for improved population health and system performance, large-scale systemic integration of digital technologies for mental healthcare has been inconsistent, and this could be attributed to a complex interaction between patient, professional, organisational, and policy barriers [15, 17–19, 20].

Given the limited scope of previous reviews, it is critical to advance evidence synthesis in this area by identifying barriers and facilitators to the implementation of digital tools in mental health systems using a multi-domain implementation framework, which can inform policies for an equitable and systemic digital transition. In this qualitative systematic review, we aim to provide a thematic synthesis of barriers and facilitators to the integration of digital technologies in mental healthcare systems to inform policy recommendations. Drawing from two established frameworks, barriers and facilitators will be mapped across implementation domains [21], organised by levels of mental healthcare systems [22], thus capturing the complexity of the mental healthcare environment and the associated impact of these multiple factors on implementation.

## Methods

The methodology used was based on the Joanna Briggs Institute (JBI) framework for systematic reviews of qualitative evidence [23]. This review is reported according to the preferred reporting items for systematic review and meta-analysis (PRISMA) [24] (Table A1, Appendix). The protocol has been published and is registered on PROSPERO (CRD42021276838) [25]. Although we originally planned to use the healthcare ecosystem approach to mental health research developed by Furst et al. [22] to categorise the identified barriers and facilitators across different domains and levels of the health care system, we incorporated an implementation framework to map identified themes onto relevant domains [21].

### Search strategy and selection criteria

The search strategy was developed in Medline, and expanded to Embase, Scopus, PsycInfo, Web of Science, and Google Scholar in consultation with a senior librarian. The searches were limited to English language peer-reviewed studies published between 1 January 2010 and 14 January 2022. The searches were designed based on the Population, Phenomena of Interest, and Context (PICo) mnemonic designed for qualitative reviews [23]. The population included all digital health technologies as defined by the WHO Global strategy on digital health 2020–2025 [9]. The phenomena of interest include all barriers and facilitators as informed by implementation science and other qualitative or mixed-methods research. The context refers to mental health systems, defined as all activities, organisations, and resources that promote, maintain or improve mental health [26]. The search syntax for each database is attached in Table A1 of the appendix.

Studies were considered eligible only if they were peer reviewed primary research articles which report qualitative data on barriers and/or facilitators to the implementation of digital tools in mental healthcare systems. Mixed method studies were included if they provided qualitative findings identifying barriers and facilitators. Studies were excluded if they were not conducted in humans, did not focus on digital technologies used for mental health issues, did not report relevant barriers or facilitators, were not peer reviewed primary research, and were not published in English.

### Study selection

Studies identified in the search were collated and deduplicated in EndNote X9, and exported to Covidence data management software for screening. Title and abstract, and full-text screening were completed separately by CB, MH and MA, and each article at both stages was independently screened by two team members. Any conflicts which occurred during screening and reviewing were resolved by consensus among all reviewers.

### Data collection and synthesis

Selected references were read in full by CB, and each item highlighted and extracted using EPPI-Reviewer Web. All included studies were charted by CB and 10% (n = 8) of them were charted a second time by MH, with 90% agreement on total codes created (95/106 codes). A new code tool was created for data extraction to perform line-by-line coding of relevant studies, with relevant quotations from each article applied to a relevant code. Information extracted included study description (e.g., study characteristics, sample, technology users, mental health disorder), and study outcomes (i.e., barriers and facilitators). The full list of variables information extracted from each study is described in Table A3 of the appendix.

The results are reported based on the Enhancing transparency in reporting the synthesis of qualitative research (ENTREQ) guidelines [27] (Table A6, Appendix). CB and ZJ performed both inductive and deductive cycles of thematic analysis, supported using EPPI-Reviewer Web. The method described by Thomas and Harden [28], including three steps, was used for thematic synthesis: (1) findings identified in the primary studies relating to barriers and facilitators to the implementation of digital technologies were coded line-by-line; subsequent studies were coded into pre-existing concepts, and new concepts were created when deemed necessary; (2) free codes were inductively organised by assigning descriptive themes based on meaning and content, with new themes added as appropriate; (3) analytical themes were constructed deductively, by organising data according to a published implementation framework. Seven implementation frameworks were tested to determine which was the best fit to the identified themes [21, 22, 29–33]. Cochrane’s framework [21] was selected because it provided an excellent fit to the data with an appropriate level of granularity to describe findings. For each domain, similar findings were aggregated and accompanied by an inclusive statement representing all the findings of the specific domain (Table A8, Appendix). Findings were also tabulated by levels of health systems described in the Healthcare Ecosystem Research in Mental Health framework [22].

### Assessment of methodological quality

Critical quality appraisal of the final articles selected was performed by ZJ and MA with disagreement solved by consensus among all reviewers, using JBI Critical Appraisal Checklist for Qualitative Research (Table A4, Appendix) [34]. Table A5 Appendix reports assessment for all included study, assigning a score (“yes”, “unclear”, or “no”) to each cell within five categories. For each category, overall assessment is based on total number of scores within specific category. Papers were not excluded on the basis of study quality, and critical appraisal was used to inform assessment of confidence in evidence, according to Grade CERQual guidelines. To provide robust policy recommendations, an indication on the level of credibility of the findings was reported, using GRADE-CERQual [35, 36]. For each domain, “no or very minor ‘, ‘minor“, ‘moderate’ and ‘serious’ concerns were assessed by CB and checked by MA, with discrepancies resolved by consensus (Table A10, Appendix).

## Results

**Fig. 1:**
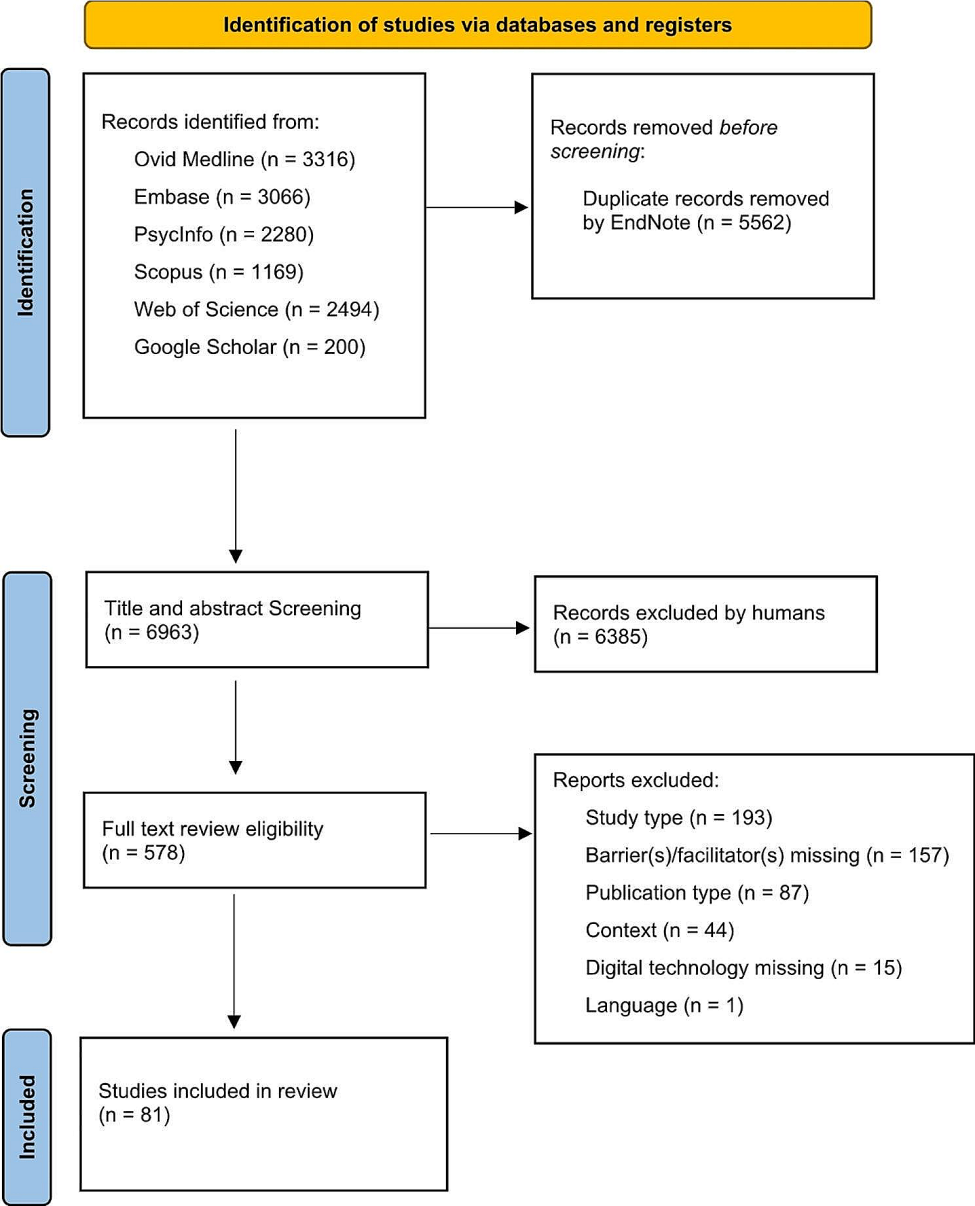
PRISMA flow diagram of included studies [24]

Of 12,525 initial references identified through database searching, 6,963 unique studies were screened for title and abstract eligibility after duplicates were removed. 81 studies were included in the qualitative synthesis (Fig. 1). The included studies were heterogenous (Table A7, Appendix). 57 studies were published after 2017 and 6 focused on more than one country. Studies were primarily conducted in high-income countries, including 22 in US, 18 each in UK and Australia. 61 studies were qualitative, with sample size ranging from 2 to 791 (median = 67.3). While 15 studies referred to digital technology in general, 19 were specifically focused on telehealth, 11 on mobile applications (apps), 8 on computerised CBT, 7 on mobile health, 6 on web-based programs, 5 on the internet of things, 4 on use of telephone and text messages, 3 on digital platforms, 2 on electronic record systems, and 1 on artificial intelligence. 58 studies focused on mental health professionals guided technologies. The majority of studies were conducted in the general population (n = 57). Other studies were conducted in specific population groups, including veterans (n = 5), Aboriginal and Torres Strait Islander people (n = 3), children (n = 3), adults (n = 2) adolescents (n = 5), students (n = 3), and 1 each in men, migrant, and refugee populations. 60 studies focused on general mental health, whilst other studies recruited participants with reference to specific disorders including for depression (n = 11), and one each for bipolar, borderline personality, eating, gambling and post-traumatic stress disorders, suicidal ideation, perfectionism, and psychosis.

**Fig. 2:**
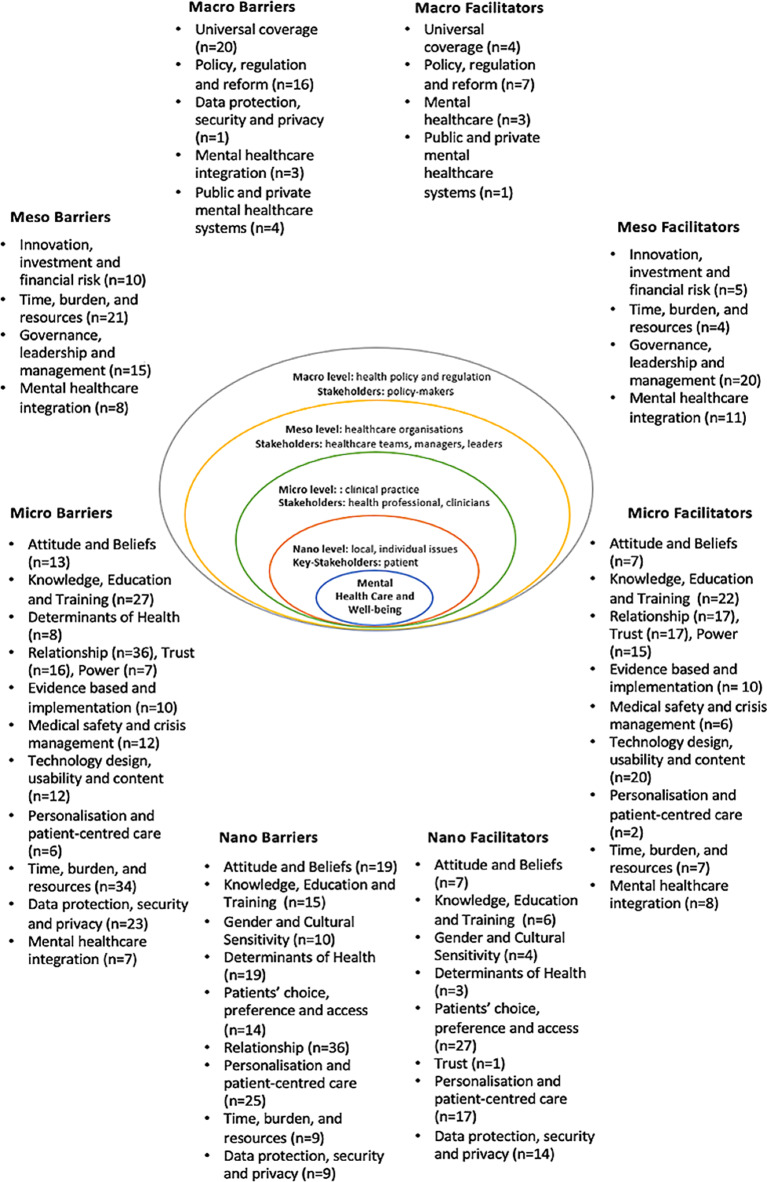
Systemic representation of barriers and facilitators to the implementation of digital health technologies across levels of mental health systems according to Furst [22]

**Fig. 3:**
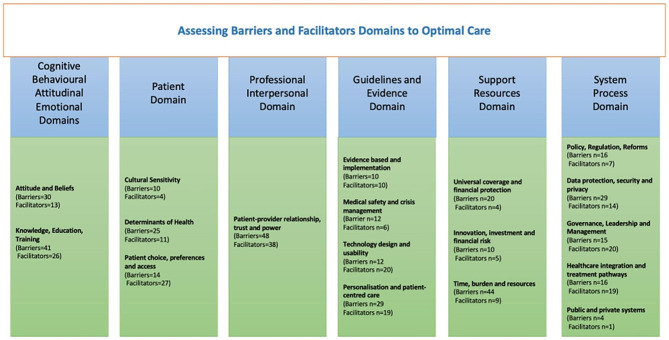
Organisation of barriers and facilitators into implementation domains according to Cochrane [21]

Findings that identified barriers and facilitators were all unequivocal and supported by primary evidence. Barriers and facilitators were identified in a framework including four healthcare system levels: (1) macro (country); (2) meso (organisation or service); (3) micro (professionals); (4) nano (patient), derived from Furst [22] (Fig. 2) and five implementation domains: (1) cognitive, behavioural, attitudinal and emotional; (2) patient; (3) professional and interpersonal; (4) guidelines and evidence; support and resources; (5) system and process, from Cochrane [21] (Fig. 3). Cognitive and behavioural, and attitudinal and emotional domains were combined in a single category for a better fit with the data. Descriptive themes supported by representative quotes for each domain and sub-domains are illustrated in Table A8 appendix.

### Cognitive, behavioural, attitudinal, and emotional domains

Sixty-six papers described barriers and facilitators across the cognitive, behavioural, attitudinal, and emotional domain, broadly divided into themes of attitude and beliefs (n = 37; nano = 24, micro = 18), and knowledge, education, and training (n = 48; nano = 19, micro = 33).

At the individual patient level, pre-existing beliefs about the effectiveness of digital interventions [37–42], lack of motivation [43–50], resistance to change [42, 51, 52], negative previous experience [53] limited patients’ willingness to use digital mental health technologies. Patients perceived digital treatments as a less rigorous way of dealing with problems [49] or reported feeling discomfort communicating emotions via technology as opposed to face-to-face [43, 54]. Patients also reported that they perceived providers using digital technologies as being less qualified compared to those providing traditional modes of delivery [37, 38]. Facilitators at the patient level included a positive perception of professional-looking technologies, which were considered to enhance treatment legitimacy [55–57], or support from an online community of peers [58]. Digital treatment also contributed to destigmatising the receipt of mental healthcare [54, 56], including in young people [59] and men [60]. Negative attitudes and beliefs around digital technology were also commonly cited barriers at the provider level, including inferior perceived quality, effectiveness, and efficiency [61–65]. Providers were also resistant to change their practice or lacked motivation to incorporate digital service provision [45, 51, 55, 66–70]. Conversely, having a positive attitude, and motivation or willingness to support integration of technology [45, 46, 71, 72], engaging with technology to avoid being left behind [53, 71], and a cultural shift to a digital mindset [73] were common facilitators to implementation for providers.

Overall, patients cited their own technological capabilities and skills as a barrier [39, 41, 47, 49, 67, 74–78]. Other barriers related to knowledge, education and training at the patient level included limited information and guidance provided by health professionals [79, 80], and a lack of awareness or knowledge about digital mental health interventions available [37, 42]. Active promotion of digital mental health technologies [46, 81, 82], and credentialling by trusted sources [41, 42, 52] were facilitators for patients. At the provider level, technological capabilities [51, 55, 65, 83–87], insufficient training, knowledge and education [50, 51, 57, 64, 73, 88, 89], and low self-confidence [53, 55, 62, 76], were cited as barriers to the use of digital mental health services, as well as a lack of awareness of available evidence-based technologies [72, 75, 81, 90], and a limited understanding of the value technologies can add for end-users [81, 91]. Other barriers include scarce or absent digital literacy, especially among older health workers [71, 81, 90, 92–94]. Access to training to acquire digital competencies was the most commonly cited facilitator to uptake at the provider level [44, 50–53, 55, 64, 66, 72, 81, 85, 89, 90, 92, 95–97]. Other facilitators include familiarity and confidence with technology [57, 71, 93], education and critical understanding of the value of technology according the patients’ needs [53], provision of comprehensive resources for clinicians and patients to introduce digital tools and understand their functions [53, 73, 89], and independent consultation with people outside the service that have previous experience with the tool [50].

### Patient

Fifty-four studies described barriers and facilitators across the patient domain including gender and cultural sensitivity (n = 11; nano = 14), socio-economic determinants of health (n = 25; nano = 22, micro = 8), and patients’ preferences for and access to digital technologies (n = 34; nano = 34). Digital technologies may not be adapted to users’ identities in terms of language [72, 73, 78, 81, 95, 98], gender [38, 60, 95], religion [38], and culture [55, 72, 85]. A lack of gender and cultural sensitivity poses a barrier by failing to meet the needs of certain population groups, for example the lesbian, gay, bisexual, transgender, intersex, queer/questioning and more (LGBTIQ+) [38], Indigenous, Aboriginal and Torres Strait Islander, and First Nations people [55, 72, 85, 95], and migrants [78, 98]. The design and content of digital mental health interventions should allow flexibility to represent gender and cultural diversity of users [59, 72, 95, 98].

Socio-economic determinants of health are the living and working conditions, which impact health outcomes and exacerbate inequalities in access to healthcare services for disadvantaged populations, including digital care [84]. Social determinants shown to act as barriers to access to digital technologies include patient’s level of education [48, 50], literacy [92, 99], and digital literacy [42, 48, 50, 52, 55, 70, 90, 92, 96, 100, 101], income and ability to pay for devices, data and internet connection [38, 52, 67, 92, 99, 101], and age [53, 55, 72, 92, 98]. Similar findings for age were reported for clinician uptake of technologies [51, 53, 72, 97, 102–104]. Free devices or apps can incentivise uptake for some patients [38, 67, 92].

Engagement with digital technologies was influenced by emotional barriers such as feeling scrutinised [76], the perception of technologies as rigid and artificial [43, 76, 80, 95], an unwillingness to spend additional time on technology after work [47, 49, 60, 105], or feeling overwhelmed by the number of digital interventions available [41, 55, 58, 101, 104]. Improved access to care was broadly shown to facilitate implementation [41, 50, 56], with the most highly cited specific accessibility measures including flexibility and availability of digital technologies and resources when needed [39, 40, 46, 47, 49, 54, 56, 60, 75, 78, 85, 88, 97, 98, 100, 103], and ease of integration into routine activities and places such as home or office [47, 49, 60, 85, 89]. Other facilitators included the ability to review materials and resources at a convenient time [49], reduced waiting times [37, 54, 74, 94], reduced costs [61, 84], enhanced choice of treatment delivery modalities [51, 54], and providing an option for those who may not seek traditional face-to-face mental health care [74].

### Professional and interpersonal domain

Fifty-eight studies described barriers and facilitators related to the professional and interpersonal domain, including relationships (n = 44; nano = 36, micro = 44), trust (n = 30; nano = 1, micro = 28), and power (n = 19; micro = 19).

The patient-provider interpersonal relationship is affected by technology use, whether it is used as a mediator, or as a substitute for, face-to-face mental healthcare. Absence of human interaction and non-verbal language, empathy, and impersonality has been cited as a barrier by both patients and providers [37, 39–44, 46, 48, 49, 51, 53, 54, 56, 61, 63, 64, 68, 69, 71, 73, 74, 76, 81, 86, 88, 93, 94, 96, 103, 106–108]. As technology cannot fully replace human interactions for mental health care [40, 49, 90, 97], some clinicians suggested that the most favourable place in therapy for digital interventions may be to complement and supplement face to face sessions [40, 45, 54, 56, 66, 72, 75, 76, 81, 90, 97, 103, 104, 109, 110], or to provide end of therapy support [67, 100], rather than to substitute completely for traditional care.

From the providers perspective, technology may be seen to intrude upon the therapeutic alliance [66, 68, 70, 72, 76, 94, 109], and trust [37, 58, 64, 100, 106, 111]. For example, some providers consider that digital delivery if care may be vulnerable to manipulation by patients [78, 79, 104, 106] (e.g., symptoms simulation). Others argue that technology facilitates the therapeutic alliance by enhancing the quality of the encounter [65, 82], working as a third-party mediator [68, 95], facilitating discussions [65, 68], improving active listening [88], and communication, coordination, and collaboration with patients [57, 88]. Technology can also facilitate access to treatment for difficult-to-reach populations, including those who are resistant to open up [41–43, 49, 50, 88, 97, 112]. Patients may feel less lonely [51]. However, there was a concern that professional boundaries may be blurred when using specific technologies such as social networks, and clinicians did not wish to appear ‘too available’ when using these social tools [40, 82, 100].

Providers can perceive the introduction of digital technologies into their practice as an imposition outside the scope of their profession, driven by external pressure and expectations rather than naturally emerging from professional choice and contextual needs [46, 50, 94]. The shift to digital technologies creates perceived job insecurity and concerns about an over-reliance on technological tools for decision making [68, 71], and a feeling of reduced need for their professional and clinical expertise [50, 71, 94, 106]. Providers are worried that responsibilities for care may be excessively shifted from the state onto patients, e.g., individualisation [40]. The most commonly cited facilitators were the empowerment of patients, increased self-reliance, patient involvement in the process of care, and improved patient-provider reciprocity [38, 40, 46, 54, 67, 72, 75, 76, 82, 94, 95, 98, 100, 104].

### Guidelines and evidence

Fifty-seven studies described barriers and facilitators across the guidelines and evidence domain including evidence-based care and implementation (n = 17; micro = 17), medical safety and crisis management (n = 16; micro = 16), technology design, usability, and content (n = 30; micro = 30), and personalisation and patient-centred care (n = 38; nano = 37, micro = 6).

Providers cited difficulty in identifying evidence-based technology for mental health, including a lack of guidelines and repositories of effective tools [62, 71, 72, 91, 94, 100, 103]. Further, difficulty measuring and monitoring outcomes for patients who were treated via digital tools, such as telephone-delivered interventions, was also cited as a barrier [51, 81, 112]. The most commonly cited facilitator was the inclusion of specific evidence-based technologies in guidelines giving clinicians evidence-based information on expected mental health outcomes e.g., improvement of symptoms [61, 66, 71, 72, 79, 81, 93, 95, 96, 106].

Providers cited barriers including inadequate risk management, unclear professional liability issues, delegation of responsibility in an emergency e.g., self-harm, suicide, or cyber bullying [40, 62, 65, 67, 82, 88, 90, 100, 103, 104, 106]. Patients stated that the presence of professional moderators on websites, and the ease of accessing help in an emergency [58] were facilitators. The presence of safety protocols, including in case of emergency [77, 97, 111], and guided use of technology, such as by offering limited therapist support alongside an online intervention [48, 100, 104] were commonly cited facilitators for health professionals.

Design problems [38, 51, 65, 68, 91], complicated technology [61], inappropriate motivational content [51], lack of flexibility [65, 68, 76], lack of interactivity [51], monotonous and repetitive content [51], absence of content personalisation options [48, 66, 68, 82, 100], and user fatigue [59, 66] were all barriers to use cited by health professionals. Attractive design [51, 52, 59, 67, 72, 78, 95, 98, 101, 109, 113], ease of use [51, 72, 79, 97], perceived usefulness [51, 55, 57, 73, 74, 90], and flexibility and portability of device the intervention is offered on [55, 72] were all major facilitators. Other cited enablers included co-production between developers, clinicians, and service users [45, 81].

Providers stated that technologies, such as apps, tend to lack customisability [55, 62, 96, 103] and the flexibility and adaptability required to provide person-centred care [45, 53, 66, 69, 70, 73, 75, 88, 90, 96, 98]. In some cases, the severity or acuity of the mental health condition [49, 61, 63, 71–73, 80, 114] or disability [62] was cited as a barrier to use of digital technologies which could not be customised. Patients also reported that digital interventions failed to take into account users’ sensory abilities [101], risk of device dependence [40, 82, 108], and may amplify feelings of social isolation for people living in remote environments [104]. Allowing tailoring and customisation of the medium, which could increase control over users’ experience according to their needs and demographic profile [37, 49, 53–56, 59, 68, 71, 82, 92, 98, 100, 103, 108, 109, 113, 115], implementation of person-centred models of care [70, 73], and inclusion of users’ preferences in digital care plans [73] were all facilitators to implementation.

### Support and resources

Fifty-one studies described barriers and facilitators across the support and resources domain including universal coverage and financial protection (n = 22; macro = 22), innovation, investment, and financial risk (n = 13; meso = 13), and time and other resources (n = 46; nano = 9, micro = 37, meso = 24).

At the policy level, digital technologies can improve access to care for the general population [82, 106], as well as people on low incomes [70], and geographically remote patients [50, 75, 83, 85, 89, 107, 110]. Barriers included poor governmental or third-party payers’ insurance entitlements to coverage [63, 94, 102], and formulary or prescribing restrictions [79, 94]. High expenditure [84], or restricted funding [50, 94, 102], third-party reimbursement, and billing complexities were all cited barriers [44, 63, 67, 81, 83, 85, 92, 94, 102, 103]. Adequate and sustainable funding [50, 69], subsidisation of digital services [72], and evolution of payment models were all facilitators [83].

At the organisational level, stakeholders face high financial risk associated with the implementation of digital technologies [91], especially in rural areas [102], characterised by high entry and maintenance costs, and rapidly changing technology [67, 81, 94, 102]. Other investment-related barriers include lack of budget for digital care [44, 71, 102, 110, 116], the high cost of technology maintenance [92]. Facilitators which can reduce the financial risk for organisations included centralised funding and resource investment [66, 79], call centres [112], and grants for innovation [83].

Integration of digital technologies can be perceived to shift additional burden of care onto both providers (e.g., additional administrative technology-related tasks) and patients (e.g., burden of self-care) [37, 41, 42, 47, 49, 56, 80, 109]. For providers, lack of time and additional workload due to the introduction of digital technologies [44, 45, 51–53, 61, 64–66, 68, 71–73, 82, 83, 86, 90, 95, 96, 102, 117] could contribute to disruption of work-life balance (e.g., less division between work and private life when working remotely with technologies) [51, 82, 84], additional bureaucracy and administrative burden associated [65, 93, 106], and labour intensive nature of scheduling of online appointments [79, 87], all cited barriers to implementation. Economic and non-economic provider incentives, including the opportunity for more flexible work for health professionals [51, 66, 94, 112], and centralised scheduling [79, 110], and management [102], were all facilitators at the provider level. At the organisational level, multiple intertwined technical and organisational barriers were cited. These included lack of stable internet connection [83], limited infrastructure in terms of devices and programs [92, 93], especially in remote settings [72, 73], maintenance [57, 101]; lack of compatibility with existing devices or systems [57, 67, 97, 101], lack of streamlining among organisational databases [71], inability to access IT support [51, 64, 117], and perceived risk of losing important data [57]. Cited organisational barriers included poor human resources and knowledge [50, 92]; staff and equipment shortages [79, 115], and personnel turnover and loss of expertise [53, 66, 68, 69, 72, 110]. Adequate resourcing, human capital, and time investment [79, 102], provision of appropriate equipment for the digital work environment [112], and technical quality [85] were cited organisational-level facilitators.

### System and process

Sixty-six studies described barriers and facilitators across the system and process domain including policy, regulation and reform (n = 16; macro = 16), data protection, security and privacy (n = 34; nano = 20, micro = 23, macro = 1), governance, leadership and management (n = 25; meso = 25), mental healthcare integration and treatment pathways (n = 30, micro = 14, meso = 18, macro = 6), and public and private mental healthcare systems (n = 4; macro = 4).

Prevailing social norms that position digital technologies as a “product” rather than a legitimate health care service [67, 94], lack of political awareness, interest and commitment, and short-term funding rather than sustained investment [51, 55, 67, 72, 94], weak leadership [81], institutional support [86], misalignment between political and clinical objectives [44], and poor marketing [94] are all barriers to implementation at the health systems level. Outdated regulation restricts or prevents the implementation of digital services [44, 62, 67, 72, 81, 103, 118]. For instance, differences in interstate licensing in some countries, and need for a referral from a GP, were commonly cited barriers to access [69, 81, 94, 102, 115]. To facilitate implementation, evidence suggests it is important to have a regulatory certification system in place to endorse credible technology solutions [40, 81, 94] and incorporate their use into guidelines and procedures [55, 72, 81, 94, 115]. Intersectoral supportive policy between sectors such as health, justice, social support with public engagement in policy development [40, 94] may allow better coordination to facilitate implementation, while public awareness through marketing will drive engagement and create acceptance and facilitate demand [40, 55, 69, 72, 81, 94, 115].

Both patients and professionals consider broad privacy issues related to the use of digital technologies [38, 51, 52, 64, 67, 81, 96, 98, 113]. Issues such as, lack of anonymity [38], absence of confidentiality [42, 43, 63, 71, 72, 87, 93, 100, 103, 115], inadequate data security and protection [54, 61, 73, 81, 101], and risk of surveillance [109] all serve as barriers. There is a perceived risk of digital devices being hacked [42, 62, 100, 101, 106], and data being lost or stolen [97, 109]. A lack of privacy at home when using remote technologies was also cited as a barrier for patients [43, 77]. Interestingly, one study cited excessive security and privacy laws as barriers to innovation in mental healthcare systems [51]. Facilitators included providing assurance of confidentiality of information such as a private way for patients to record information which is considered more secure than hand-written notes [41, 43, 51, 56, 58, 78, 98, 100, 113]. Relative anonymity compared to face-to-face sessions is also a facilitator for some people [43, 51, 52, 56, 58, 78, 98, 100, 113].

Lack of leadership and support from management [44, 51, 55, 110], absence of a long term organisational strategy and resources to implement change [44, 50, 68, 69, 71, 73, 83, 115], and staff resistance to innovate [37, 69, 94] were commonly cited barriers. Staff-related barriers also included absence of communication and collaboration among colleagues [50, 51, 95, 104, 110]. Facilitators include leaders who believe in innovation and drive implementation [45, 51, 66, 67, 83], enthusiastic, supportive and accountable managers [53, 55, 72, 85, 110, 117], organisational policies and procedures [71], positive learning climate [67]. Other cited facilitators included presence of an internal facilitation team [55] including project managers [83], and ‘champions’ of digital technology interventions within organisations across administration [83], clinicians [52, 83, 112], and IT [53, 66, 79]. Collaboration, communication, support and promotion by colleagues [50, 55, 69, 89], feeling part of a team [110], and opportunities for professional development for staff [72] facilitate technology integration. Organisational belief that the technology will deliver better care that in turn stimulates a drive for radical change [46] was also cited as a facilitator, while others stated that hybrid [94] and staged [55, 72] approaches are preferable for innovation change.

Providers frequently perceive a lack of fit of digital technologies with existing mental health practice and values [117], including difficulty in understanding patients’ symptoms via remote care [100], quantifying feelings [76], tailoring homework [86, 96], providing feedback [69], and monitoring patient use of digital tools [75]. Ease of integration into existing workflow [45, 66, 72, 79, 95], the ability to monitor patient progress [57, 76, 88], and store protocol information and patients’ homework [57] were all cited as facilitators. From an organisational perspective, a lack of integration of digital technologies into existing treatment pathways [45, 48, 65, 79, 94, 117], lack of continuity of care [70] and poor or absent cross-system communication between digital tools and existing clinic information systems [73] were barriers to implementation. Conversely, technology can also support providers’ adherence to treatment protocols [45, 57, 68, 75, 76, 109]. Adoption of a stepped-care approach and system interoperability [50, 52, 59, 60, 76, 115] were also facilitators. At a system level, a lack of health and social system integration [94, 110], and fragmented provision of care [43] were barriers, whilst systemic integration of digital technology into broader systems [38, 69, 102] was a facilitator.

Different barriers to implementation exist between public and private systems. These include lack of integration between public and private actors more broadly [94], differential policies on funding, billing and coverage [51, 102], restrictions on the use of digital technologies in public systems compared with the private sector [53], the choice to substitute or complement traditional services with digital treatment in private sector compared to public [51], and a lack of uniform coverage of services across public and third-party payers [102]. Absence of involvement of all stakeholders such as academics, health providers, end users, and private sector industry in decision making process [94] were further barriers to implementation. Public and private partnership [94] is a facilitator for successful implementation.

Identified facilitators are used for the formulation of policy solutions for each domain and level in Table 1.

**Table 1:** Policy recommendations to facilitate systemic implementation of digital technologies in mental healthcare system

Domains	Healthcare system levels			
	Nano– Patient	Micro - Professionals	Meso - Organisations	Macro- Policy
**Cognitive /Behavioural, Attitudinal, and Emotional**	• De-stigmatization around mental healthcare and legitimation of digital technologies via policies targeted to specific population groups, particularly those most likely to experience negative attitude and beliefs e.g., men, young people • Active promotion of digital technologies by trusted sources and guidance form health professionals	• Policies which promote clinicians’ attitudinal and behavioral shift toward accepting and providing digital mental healthcare • Education, training, and resources targeted to specific provider groups according to degree of digital skills e.g., older workers, to improve digital literacy of mental health professionals	**-**	**-**
**Patient**	• Digital health interventions should account for population diversity in terms of gender, religious and cultural identities • Consider the interplay between characteristics such as social, economic, and gender factors, and digital literacy as determinants of health to improve access to digital technologies in mental healthcare • Balance trade-offs between improved choice in digital mental care options, efficiency gains in integrating digital technologies, and equitable access for vulnerable groups e.g., providing free access to digital tools for certain population groups • Limiting choice on the market to a set of high quality and safe options of technologies to avoid excessive choice burden and infodemic	**-**	**-**	**-**
**Professional and Interpersonal Domain**	-	• Guidelines and training to build digital patient-provider relationship based on trust, transparent communication, and professional boundaries • Digital technologies as transitional, complementary object rather than a substitute to traditional care through guided use of technology under professional supervision • Balance patients’ empowerment through involvement in self-care and clinical expertise to guide safe use of technologies and avoid excessive burden of self-care on patients	-	-
**Guidelines and Evidence**	• Ease of use and perceived usefulness are drivers and need to be assessed case-by-case basis for tailored interventions according to demographics, epidemiological profile, and sensory ability or skills e.g., severity of illness • Co-design process of digital technologies and implementation to adopt person-centered view	• Digital health interventions should be based on guidelines, protocol, informed by evidence-based outcomes • Establishment of safety protocol to use with remote digital mental health interventions in case of self-harm • Digital technologies should provide safe, appropriate, and flexible content, on portable devices	-	-
**Support and resources**	• Supporting patients’ and tackling their difficulties in time-management and self-care burden in case of unguided use of technologies	• Rewarding additional clinical and administrative burden shifted on clinicians due to integration of technologies with economic and non-economic incentives and flexible work arrangements, to avoid burn-out	• Provide adequate conditions for healthcare organizations to innovate, such as adequate financing through grants for innovation, risk management to reduce risk related to innovation (e.g., reducing fragmentation and pooling financial risk) • Provide adequate digital work environment and technical assistance to clinicians that experience technical problems with technologies	• Digital technologies should contribute to achievement of SDGs 3: health and well-being, including universal mental health coverage tackling population, services and costs covered; implementation policies should (directly or indirectly) support these aims
**System and Process**	-	• Establishing guided implementation pathways for digital mental healthcare interventions into existing workflow and practices to ensure continuity of care	• Enthusiastic and accountable leaders and managers • Organization-based multidisciplinary facilitation teams: clinical, administrative, and technical skills • Teamwork and staff development pathways • Stepped-care approach to integrate digital technologies	• Reforms to bring political and policy awareness, adequate economic models, and updated regulation for digital technologies to expand access to mental healthcare • Transparency of IT privacy policies to ensure confidentiality of personal information and anonymity for patients • Systemic digitalization of healthcare system to improve systems inter-operability • Involvement of public, private sectors, and patients in participatory policy decision-making • Pursuing public-private partnership to innovate, balancing public interest and private profit, and sharing risks and rewards

## Risk of bias and confidence in evidence

Study quality assessment revealed that, on theoretical basis category, 18 studies scoring low and 7 medium quality. On the method category, 1 (˜ 1%) study was low and 2 medium quality. On research influence, 40 studies scored low and 32 medium quality. In the participants category, studies were assessed as 18 medium and 4 low quality. Finally, only 1 low and 5 medium quality studies in the result category (Table A5 Appendix). We did not exclude studies based on quality, however results should not be severely affected by low quality studies, as the synthesis of results for each domain was not exclusively supported by low quality studies for any domain. This is highlighted in the credibility assessment (Table A10 Appendix). This assessment, using Grade CERQual [35, 36], suggests that all domains of barriers and facilitators presents ‘no or very minor concern’, except for four domains that scored ‘minor concern’.

## Discussion

This systematic synthesis of qualitative evidence aimed to identify a range of barriers and facilitators to the systemic integration of digital technologies in mental healthcare systems, and classify them into implementation domains, across levels of the health system. The identified barriers and facilitators mapped to all domains of Cochrane’s evidence-practice gap framework, which provides sufficient granularity to inform stakeholder-targeted policies and tailored solutions to overcome barriers to the implementation of digital technologies in mental health systems. Simultaneously, they support a transition toward more equitable and efficient digital mental healthcare systems. The findings also highlight the importance of interaction, engagement, and collaboration between different public and private stakeholders to bring systemic change across different and interdependent levels of the mental healthcare system [22].

Driving change in mental health systems poses challenges due to structural stigma, which creates barriers impeding policy advancements, decreasing public demand for necessary actions, and limiting policymakers’ awareness of viable policy alternatives [119]. There is a disproportionate allocation of resources in comparison to the epidemiological, economic, and social burdens posed by mental health issues, leading to caps on benefits and lower reimbursement rates [120]. This is compounded by limited governmental expenditure, typically falling below 2% of the global median of health expenditure, allowing the persistence of structural issues in mental health care financing [121]. Such underinvestment contributes to shortages of health professionals and the corresponding skill mix required to address the increasingly complex needs of patients, particularly those affected by multimorbidity [122]. Globally, there exists a shortage of mental health-trained health workers, with a median of 9 per 100,000 population and significant disparities in access across income brackets [121]. These systemic barriers exacerbate the underdiagnosis and undertreatment of patients affected by mental health issues [123].

The integration of digital technologies into mental health systems has the potential to narrow the gap in mental health diagnosis and treatment. A significant amount of literature has been published regarding barriers and facilitators to implementing digital technologies for mental health. However, previous studies focused on single digital technologies [51, 86], specific digital treatments [56], or individual actors [16, 37] within the health system. While offering valuable insights into challenges and solutions to the effecting implementation of technologies, health system change proves to be complex [124]. There is a general lack of literature taking a systemic view, which can provide more comprehensive insights into the processes of implementation, transformation, and digital transition in mental health systems. For this reason, we conducted a systematic review and analysis using a system-wide perspective to the implementation of digital technologies in mental health systems, entailing views of different actors within the health system organized into relevant domains. Such a system-wide approach has previously been acknowledged for its ability to identify significant implications on overarching health system outcomes and value creation [125]. Our framework, cross-tabulating levels of health systems with implementation domains, offers clear lessons to policymakers to implement effective reforms at all levels for improving overall population mental health and well-being.

**At the patient (nano) level**, patient, and guidelines and evidence implementation domains were the most prevalent for the implementation of digital technologies for mental health. Challenges with the adoption and reach of digital health innovations arise due to significant gaps in the evidence-to-practice cycle. Whilst some digital technologies offer an efficient and effective standardised treatment for a population, guidelines should incorporate a degree of flexibility to develop personalised care according to most recent evidence. Implementation of these interventions, including development of policies and guidelines, should be driven by a person-centred approach to be assessed by professionals on case-by-case basis, considering population diversity including gender, class, ethnicity, health status, preferences, and disability. Digital transformations are shaped by and embedded into particular social and economic dynamics. Despite the increased access and choice of treatment which digital technologies may offer, only certain population groups may benefit from it if population heterogeneity is not considered. This is in line with previous research that found implementation of digital health as a leading factor of inequalities in the distribution of healthcare resources when this failed to be considered [126], as well as evidence of a rapid uptake of culturally competent health apps for racial minorities in the US [127]. A lack of representation in the development of digital interventions may create biased designs and algorithms [128], hampering the opportunities that digital health may offer to alleviate mental health disparities among marginalised populations [129]. Policy frameworks should consider intersectionality to tackle and prevent inequities in digital health [128]. Digital health will be affected by the same social determinants as other health processes and outcomes, and should be deployed accordingly; taking into account patient heterogeneity, digital literacy and access, and offering adaptability will help to address disparities [8]. To facilitate a patient-centred approach and enhance patients’ experiences, co-design processes are indicated as a feasible solution for incorporating the needs and requirements of end-users to provide tailored solutions, and incorporating lived experience [130–132]. In these co-design processes, it is crucial to avoid underrepresentation and exclusion of vulnerable groups [131], and to utilise a framework that elicits the needs of end-users, and tailors proven digital innovations to meet these needs.

**At the professional and interpersonal (micro) level**, knowledge, education, and training emerged as the principal domain facilitating the use of evidence-based technologies. Our review confirms the findings from previous research which found that poor digital literacy in mental health professionals was a significant barrier to the implementation of technologies in their practice [8, 17]. Therefore, it is crucial to create policies which enable a digitally literate workforce. The latter has been included as a key priority by the WHO and many governments in their national digital health plans e.g., UK [133], Australia [134], and Italy [135]. To achieve this, a significant investment must be directed towards the support and resources domain, as argued by Feijt et al. [90]. Investments in financial, human, and technical resources are essential to implement a digital transition and avoid worker burn-out. Economic (e.g., payments) and non-economic (e.g., awards) incentives for providers can play a key role, as they drive demand for digital technologies. Care providers have very specific skillsets which are vital for facilitating the shift to digital mental healthcare. Promoting shared decision-making and an awareness of information asymmetries and power dynamics between patients and providers were important facilitators at the patient-professional interpersonal domain. The need for clinical expertise should not be underestimated, especially in primarily unregulated digital technology markets, which are characterised by technologies with varying quality and safety. While studies on professional guided technologies were prevalent in our review, additional evidence is needed on the use of unguided technologies. Unguided use of mental health technology can create serious practical and ethical issues for patients, including challenges to choose a safe and effective app among the multitude currently available [108], and pressure associated with caring for one’s own mental health development [136] which can also reduce external help-seeking behaviours and increase chances of suicidal behaviours [137]. Existing provider skillsets can be leveraged to ensure the implementation of digital mental health technologies is equitable and efficacious.

**At the organisational and clinical (meso) level**, system and process, and support and resources, were the most relevant implementation domains for a digital transition in mental healthcare systems. Digital interventions should be tailored around the mental health problem treated. Stepped-care models, aligning intensity of digital health interventions to the severity of mental health disorders, should be followed to support sustainable and effective long-term implementation [138] as reported by previous systematic reviews [139, 140]. Beyond necessary fundamental clinical considerations, digital health transition should be embedded in organisational structures in a participatory process that involves multidisciplinary teams of workers e.g., clinicians, human resource managers, administrative personnel, and IT experts. For example, alongside fundamental clinical expertise, leaders and managers significantly contribute to long-term capacity building for implementing digital technologies at the organisational level, increasing the likelihood of sustained investment, and fostering team building and development [141]. Themes grouped under the support and resource domain highlighted that both financial investment, multidisciplinary facilitation teams and trainings are priorities to enable the integration of digital innovation, which should be maintained in the post-implementation period. However, barriers to innovation tend to dominate the healthcare sector generally, which represents a non-contestable market, including the need for a large up-front investment and difficulty measuring cost-effectiveness. The successful implementation of healthcare innovations is challenging, and relies on effective stakeholder cooperation in a regulated environment [142]. Therefore, institutionalizing infrastructure, involvement of different stakeholders, and strategic planning are vital for sustained access to cost-effective interventions. Practical guidelines include government- or organisation-wide digital standard framework, and the use of implementation roadmaps, and policy oversight frameworks [143, 144]. Innovation grants, with a mechanism to share risk and rewards for innovation between public and private actors, should stimulate innovation to create public value [145]. Examples of such facilitation in digital health can be seen in the Digital Health Centre of Excellence or the eHealth Hub Platform recently established by the US government [146] and European Union [147] respectively, which aim to advance digital healthcare by facilitating synergies between public and private stakeholders and fostering responsible and high-quality digital health innovation. Enabling appropriate funding mechanisms and teams across organisations will help to address implementation issues at the organisational level. 

**At policy (macro) level**, barriers and facilitators to the implementation of digital technologies in mental health systems were broadly related to the three dimensions of universal health coverage (UHC): population covered; services included; and proportion of costs directly shared by individuals [148], as emphasised in the support and resource implementation domain. For instance, during the COVID-19 emergency, access to mental healthcare pivoted to rely heavily on the use of digital technologies [39, 43, 107]. However, existing coverage regulation, health professional payments, and reimbursement policies were not necessarily tailored to digital healthcare, which limited access in some cases [39, 43, 107]. Post-pandemic, it will be particularly important to address barriers to digital mental health coverage by considering financial and regulatory barriers. Financing considerations are particularly relevant in scarce resource settings i.e., low- and middle-income countries [149, 150], and for individuals and in settings which may otherwise lack coverage. In the pathway toward achieving universal health coverage, recognizing that digital tools play an important role in improving public mental health and well-being and financing them accordingly will assist in meeting a key objective of SDG 3 [8]. Regarding the system and process domain, consumers were concerned about privacy policies, inadequate government legislation on data security, and use of information by private companies when it comes to mental health-related confidential information, in line with previous research [100, 151]. Relevant policies should prioritise the highest standard of protection of health data and digital rights, and arrangements such as laws, regulation and governance play a key-role in shaping the digital health eco-system [8].

Overall reforms should be driven by public purposes and not private profit [8]. The involvement of a range of interested parties, including governments, private sector, and civil society in creating collaborative digital health policy will promote successful reforms toward integration of digital technologies in mental healthcare systems, potentially improving public mental health and avoiding the exacerbation of health inequities [8, 9].

## Conclusion and policy implications

To our knowledge, this study is the first review to provide a framework categorising systemic barriers and facilitators to the implementation of digital technologies across levels of mental healthcare systems. There is a complex interaction between barriers and facilitators by domains and levels of the health care system, that affects the implementation of digital healthcare. Overall, the identified barriers and facilitators highlight the importance of patient-centred care, health equity considerations, patient and provider education, collaborative policymaking between organisations and governments, and policy directives and reforms to support change and innovation, which are evidence-based but adaptable to local contexts. Our systematic review had several limitations. Firstly, we acknowledge that relevant non-English and emerging grey literature might be missing, including reports by organisations and governments. Secondly, results were primarily drawn from experiences of high-income countries; therefore, we acknowledge that barriers and facilitators to the implementation of digital technologies in middle- and low-income countries are likely to be underrepresented in this review. Finally, the breadth of this review, which focused on high-level barriers and facilitators to the implementation of all digital mental health interventions and supports across levels of the health system, regardless of specific mental health disorder, may have neglected to identify situation specific factors. Nevertheless, this was a thorough and systematic assessment of the broad spectrum of health services, and the unique needs of different levels of the mental health system.

This study demonstrated that, despite the potential of digital technologies to improve equity and efficiency of mental healthcare systems, a complex array of barriers hampers their implementation. However, we found clear evidence for facilitators to implementation, which may be leveraged to enable a sustainable and long-term digital mental health transition. Decision-makers should consider needs and preferences of single agents in mental health systems, whilst simultaneously adopting a systemic view considering interactions between agents at various levels of the health system, with the aim of overcoming the identified barriers. Policymakers will succeed in this effort only if they will consider different strategies across various implementation domains and levels of the health system as facets of an overarching approach, and not as independent and disconnected dimensions, to facilitate systemic change. The availability of effective technologies to treat mental health is not sufficient for articulating successful policies, because they relate to organisational arrangements of health systems [152]. Policies need to be informed by frameworks that incorporate a health system perspective and consider complex interrelations between its components [152]. The recommendations from this study will support the implementation of digital mental health services and strengthen mental health systems into the future. Future research may focus on nuanced aspects of care, such as specific barriers and facilitators associated with type and severity of mental illness, high and low resource settings, guided and unguided technologies, service provider or organisation type, and policymakers.

### Electronic supplementary material

Below is the link to the electronic supplementary material.

**Supplementary Material 1:** Appendix

## Data Availability

The datasets used and/or analysed during the current study are available from the corresponding author on reasonable request.
