# Supplementary material for: Barriers and facilitators to the implementation of digital technologies in mental health systems: a qualitative systematic review to inform a policy framework
Source: BMC Health Serv Res. 2024 Feb 26;24:243. doi: 10.1186/s12913-023-10536-1 (PMC10898174; doi:10.1186/s12913-023-10536-1)
Supplement: Supplementary file 1 — Supplementary Material 1: Appendix [file 12913_2023_10536_MOESM1_ESM.docx]

**Appendix**

*Table A1: PRISMA Checklist*

*From: Page MJ, McKenzie JE, Bossuyt PM, Boutron I, Hoffmann TC, Mulrow CD, et al. The PRISMA 2020 statement: an updated guideline for reporting systematic reviews. BMJ 2021;372:n71. doi: 10.1136/bmj.n71*

| **Section and Topic** | **Item #** | **Checklist item** | **Location where item is reported** |
| --- | --- | --- | --- |
| **TITLE** | | |  |
| Title | 1 | Identify the report as a systematic review. | Page 1 |
| **ABSTRACT** | | |  |
| Abstract | 2 | See the PRISMA 2020 for Abstracts checklist. |  |
| **INTRODUCTION** | | |  |
| Rationale | 3 | Describe the rationale for the review in the context of existing knowledge. | Page 1 |
| Objectives | 4 | Provide an explicit statement of the objective(s) or question(s) the review addresses. | Page 1 |
| **METHODS** | | |  |
| Eligibility criteria | 5 | Specify the inclusion and exclusion criteria for the review and how studies were grouped for the syntheses. | Page 2 |
| Information sources | 6 | Specify all databases, registers, websites, organisations, reference lists and other sources searched or consulted to identify studies. Specify the date when each source was last searched or consulted. | Page 2 |
| Search strategy | 7 | Present the full search strategies for all databases, registers and websites, including any filters and limits used. | Appendix, Table 3 |
| Selection process | 8 | Specify the methods used to decide whether a study met the inclusion criteria of the review, including how many reviewers screened each record and each report retrieved, whether they worked independently, and if applicable, details of automation tools used in the process. | Page 3 |
| Data collection process | 9 | Specify the methods used to collect data from reports, including how many reviewers collected data from each report, whether they worked independently, any processes for obtaining or confirming data from study investigators, and if applicable, details of automation tools used in the process. | Page 2 |
| Data items | 10a | List and define all outcomes for which data were sought. Specify whether all results that were compatible with each outcome domain in each study were sought (e.g. for all measures, time points, analyses), and if not, the methods used to decide which results to collect. | Page 2 |
|  | 10b | List and define all other variables for which data were sought (e.g. participant and intervention characteristics, funding sources). Describe any assumptions made about any missing or unclear information. | Appendix, Table 4 |
| Study risk of bias assessment | 11 | Specify the methods used to assess risk of bias in the included studies, including details of the tool(s) used, how many reviewers assessed each study and whether they worked independently, and if applicable, details of automation tools used in the process. | Page 3 |
| Effect measures | 12 | Specify for each outcome the effect measure(s) (e.g. risk ratio, mean difference) used in the synthesis or presentation of results. | N/A |
| Synthesis methods | 13a | Describe the processes used to decide which studies were eligible for each synthesis (e.g. tabulating the study intervention characteristics and comparing against the planned groups for each synthesis (item #5)). | Page 2 |
|  | 13b | Describe any methods required to prepare the data for presentation or synthesis, such as handling of missing summary statistics, or data conversions. | N/A |
|  | 13c | Describe any methods used to tabulate or visually display results of individual studies and syntheses. | Page 2 |
|  | 13d | Describe any methods used to synthesize results and provide a rationale for the choice(s). If meta-analysis was performed, describe the model(s), method(s) to identify the presence and extent of statistical heterogeneity, and software package(s) used. | Page 2 |
|  | 13e | Describe any methods used to explore possible causes of heterogeneity among study results (e.g. subgroup analysis, meta-regression). | Page 5 |
|  | 13f | Describe any sensitivity analyses conducted to assess robustness of the synthesized results. | Page 16 |
| Reporting bias assessment | 14 | Describe any methods used to assess risk of bias due to missing results in a synthesis (arising from reporting biases). | Page 16, Appendix Table 6 |
| Certainty assessment | 15 | Describe any methods used to assess certainty (or confidence) in the body of evidence for an outcome. | Page 16 |
| **RESULTS** | | |  |
| Study selection | 16a | Describe the results of the search and selection process, from the number of records identified in the search to the number of studies included in the review, ideally using a flow diagram. | Page 4, Figure 1 |
|  | 16b | Cite studies that might appear to meet the inclusion criteria, but which were excluded, and explain why they were excluded. | Figure 1 |
| Study characteristics | 17 | Cite each included study and present its characteristics. | Page 4 |
| Risk of bias in studies | 18 | Present assessments of risk of bias for each included study. | Appendix Table 6 |
| Results of individual studies | 19 | For all outcomes, present, for each study: (a) summary statistics for each group (where appropriate) and (b) an effect estimate and its precision (e.g. confidence/credible interval), ideally using structured tables or plots. | Page 4 |
| Results of syntheses | 20a | For each synthesis, briefly summarise the characteristics and risk of bias among contributing studies. | Page 16 |
|  | 20b | Present results of all statistical syntheses conducted. If meta-analysis was done, present for each the summary estimate and its precision (e.g. confidence/credible interval) and measures of statistical heterogeneity. If comparing groups, describe the direction of the effect. | Page 8-16 |
|  | 20c | Present results of all investigations of possible causes of heterogeneity among study results. | Page 4, Table |
|  | 20d | Present results of all sensitivity analyses conducted to assess the robustness of the synthesized results. | Table 8 |
| Reporting biases | 21 | Present assessments of risk of bias due to missing results (arising from reporting biases) for each synthesis assessed. | N/A |
| Certainty of evidence | 22 | Present assessments of certainty (or confidence) in the body of evidence for each outcome assessed. | Page 16 |
| **DISCUSSION** | | |  |
| Discussion | 23a | Provide a general interpretation of the results in the context of other evidence. | Page 20 |
|  | 23b | Discuss any limitations of the evidence included in the review. | Page 21 |
|  | 23c | Discuss any limitations of the review processes used. | Page 21 |
|  | 23d | Discuss implications of the results for practice, policy, and future research. | Page 20-21 |
| **OTHER INFORMATION** | | |  |
| Registration and protocol | 24a | Provide registration information for the review, including register name and registration number, or state that the review was not registered. | Page 2 |
|  | 24b | Indicate where the review protocol can be accessed, or state that a protocol was not prepared. | Page 2 |
|  | 24c | Describe and explain any amendments to information provided at registration or in the protocol. | Page 2 |
| Support | 25 | Describe sources of financial or non-financial support for the review, and the role of the funders or sponsors in the review. | Page 23 |
| Competing interests | 26 | Declare any competing interests of review authors. | Page 23 |
| Availability of data, code and other materials | 27 | Report which of the following are publicly available and where they can be found: template data collection forms; data extracted from included studies; data used for all analyses; analytic code; any other materials used in the review. | Page 23 |

Table A2: Search Syntax

Database: Ovid MEDLINE(R) and Epub Ahead of Print, In-Process, In-Data-Review & Other Non-Indexed Citations and Daily <1946 to January 12, 2022>

Search Strategy:

--------------------------------------------------------------------------------

1 telemedicine/ or telerehabilitation/ (32554)

2 (telemedicine or tele-medicine or telemental or tele-mental or telehealth or tele-health or telepsychiatr* or tele-psychiatr*).ti,ab,kw,kf. (26570)

3 biomedical technology/ or health technolog*.ti,ab,kw,kf. (15635)

4 (digital adj2 (health or technolog* or revolution or intervention*)).ti,ab,kw,kf. (9823)

5 emerging technolog*.ti,ab,kw,kf. (7335)

6 (ehealth or e-health or e-mental or e-therap* electronic health or mhealth or m-health or mobile health).ti,ab,kw,kf. (18377)

7 mobile applications/ (9313)

8 (app or apps or app-based or appbased).ti,ab,kw,kf. (36001)

9 (mobile adj2 application*).ti,ab,kw,kf. (6012)

10 internet/ or internet-based application/ (78123)

11 (website* or web-site* or web-based or internet or internet-based or online or on-line).ti,ab,kw,kf. (291305)

12 1 or 2 or 3 or 4 or 5 or 6 or 7 or 8 or 9 or 10 or 11 (419692)

13 mental health/ (50015)

14 mental disorders/ (171576)

15 (mental health or mental healthcare or behavio?ral health or mental disorder* or mental illness*).ti,ab,kw,kf. (253144)

16 mental health services/ or community mental health services/ (54949)

17 13 or 14 or 15 or 16 (390370)

18 "delivery of health care"/ or health care reform/ or health services accessibility/ or (reform or reforms or reforming).ti,ab,kw,kf. (236332)

19 Health Policy/ or (policy or policies).ti,ab,kw,kf. (340862)

20 health services research/ (38014)

21 global health/ or (global or globally).ti,ab,kw,kf. (555430)

22 economics/ or "cost and cost analysis"/ or health care costs/ or health expenditures/ or (cost* or expenditure* or financ* or reimbursement* or economic* or austerit* or fund or funded or funding or underfund* or fragment* or welfare).ti,ab,kw,kf. (1619628)

23 (deliver* adj3 (care or healthcare or system*)).ti,ab,kw,kf. (144217)

24 (healthcare system* or health care system* or health system* or mental healthcare system* or "mental health care system*").ti,ab,kw,kf. (137546)

25 "health service needs and demand"/ or needs assessment/ or (assess* adj1 need*).ti,ab,kw,kf. (38678)

26 18 or 19 or 20 or 21 or 22 or 23 or 24 (2654148)

27 25 and 26 (13360)

28 26 or 27 (2654148)

29 12 and 17 and 28 (6752)

30 ((implement* or innovat* or integrat* or uptake* or up-tak* or accept* or scal* up or scal*-up or up-scal* or adher* or promot* or adopt* or engag* or improvement* or intervention*) and (barrier* or impede or impediment* or facilitat* or challeng* or solution* or driver* or optimi* or diffus* or infus* or success* or limitation* or strateg* or approach* or hinder* or obstacle* or hurdle* or opportunit* or enabl* or sustain* or operational* or adapt* or capacit* or polic*)).ti,ab,kw,kf. (2425186)

31 29 and 30 (3562)

32 limit 31 to yr="2010 -Current" (3414)

33 limit 32 to (comment or editorial or letter or news) (39)

34 32 not 33 (3375)

35 limit 34 to english language (3316)

***************************

Database: Embase <1947 to present>

Search Strategy:

--------------------------------------------------------------------------------

1 telemedicine/ or telerehabilitation/ or telepsychiatry/ or teletherapy/ (38631)

2 (telemedicine or tele-medicine or telemental or tele-mental or telehealth or tele-health or telepsychiatr* or tele-psychiatr*).ab,kw,ti. (34603)

3 medical technology/ or health technolog*.ti,ab,kw. (47969)

4 (digital adj2 (health or technolog* or revolution or intervention*)).ti,ab,kw. (9556)

5 emerging technolog*.ti,ab,kw. (8432)

6 (ehealth or e-health or e-mental or e-therap* electronic health or mhealth or m-health or mobile health).ti,ab,kw. (19109)

7 mobile application/ (16484)

8 (app or apps or app-based or appbased).ti,ab,kw. (48491)

9 (mobile adj2 application*).ti,ab,kw. (7054)

10 exp internet/ or internet-based application/ (119359)

11 (website* or web-site* or web-based or internet or internet-based or online or on-line).ti,ab,kw. (398161)

12 1 or 2 or 3 or 4 or 5 or 6 or 7 or 8 or 9 or 10 or 11 (594573)

13 mental health/ (169242)

14 mental disease/ (267604)

15 (mental health or mental healthcare or behavio?ral health or mental disorder* or mental illness*).ti,ab,kw. (306643)

16 mental health care/ or community mental health service/ (31421)

17 13 or 14 or 15 or 16 (563457)

18 health care delivery/ or health services accessibility/ or (reform or reforms or reforming).ti,ab,kw. (287363)

19 Health care policy/ or (policy or policies).ti,ab,kw. (482725)

20 health services research/ (35131)

21 global health/ or (global or globally).ti,ab,kw. (678317)

22 economics/ or "cost benefit analysis"/ or health care cost/ or (cost* or expenditure* or financ* or reimbursement* or economic* or austerit* or fund or funded or funding or underfund* or fragment* or welfare).ti,ab,kw. (2289845)

23 (deliver* adj3 (care or healthcare or system*)).ti,ab,kw. (172755)

24 (healthcare system* or health care system* or health system* or mental healthcare system* or "mental health care system*").ti,ab,kw. (185297)

25 health care need/ or needs assessment/ or (assess* adj1 need*).ti,ab,kw. (64684)

26 18 or 19 or 20 or 21 or 22 or 23 or 24 (3574579)

27 25 and 26 (28658)

28 26 or 27 (3574579)

29 12 and 17 and 28 (8821)

30 health care planning/ or integrated health care system/ or ((implement* or innovat* or integrat* or uptake* or up-tak* or accept* or scal* up or scal*-up or up-scal* or adher* or promot* or adopt* or engag* or improvement* or intervention*) and (barrier* or impede or impediment* or facilitat* or challeng* or solution* or driver* or optimi* or diffus* or infus* or success* or limitation* or strateg* or approach* or hinder* or obstacle* or hurdle* or opportunit* or enabl* or sustain* or operational* or adapt* or capacit* or polic*)).ti,ab,kw. (3302833)

31 29 and 30 (4486)

32 limit 31 to yr="2010 -Current" (4243)

33 limit 32 to (abstract report or books or "book review" or chapter or conference abstract or conference paper or "conference review" or editorial or letter or note) (1129)

34 32 not 33 (3114)

35 limit 34 to english language (3066)

***************************

Database: APA PsycInfo <1806 to January Week 1 2022>

Search Strategy:

--------------------------------------------------------------------------------

1 exp Telemedicine/ or exp Telerehabilitation/ (10599)

2 (telemedicine or tele-medicine or telemental or tele-mental or telehealth or tele-health or telepsychiatr* or tele-psychiatr*).ti,ab. (4314)

3 Technology/ or health technolog*.ti,ab. (41056)

4 (digital adj2 (health or technolog* or revolution or intervention*)).ti,ab. (4079)

5 emerging technolog*.ti,ab. (1264)

6 (ehealth or e-health or e-mental or "e-therap* electronic health" or mhealth or m-health or "mobile health").ti,ab. (3077)

7 exp Mobile Applications/ (1567)

8 (app or apps or app-based or appbased).ti,ab. (8916)

9 (mobile adj2 application*).ti,ab. (1646)

10 exp Internet/ (30602)

11 (website* or web-site* or web-based or internet or internet-based or online or on-line).ti,ab. (152702)

12 1 or 2 or 3 or 4 or 5 or 6 or 7 or 8 or 9 or 10 or 11 (206028)

13 exp Mental Health/ (77008)

14 Mental Disorders/ (90483)

15 ("mental health" or "mental healthcare" or "behavio?ral health" or "mental disorder*" or "mental illness*").ti,ab. (270580)

16 exp mental health services/ or exp Community Mental Health Services/ (44268)

17 13 or 14 or 15 or 16 (331324)

18 exp Health Care Services/ or exp Health Care Delivery/ or exp Health Care Psychology/ or exp Health Care Reform/ or exp Health Care Access/ or exp Health Service Needs/ or (reform or reforms or reforming).ti,ab. (264437)

19 exp Health Care Policy/ or (policy or policies).ti,ab. (180973)

20 evidence based practice/ (19390)

21 exp Global Health/ (2754)

22 economics.mp. or health care costs/ or "costs and cost analysis"/ or exp Health Insurance/ or "health expenditures".mp. or (cost* or expenditure* or financ* or reimbursement* or economic* or austerit* or fund or funded or funding or underfund* or fragment* or welfare).ti,ab. (364884)

23 (deliver* adj3 (care or healthcare or system*)).ti,ab. (17065)

24 public health services/ or ("healthcare system*" or "health care system*" or "health system*" or "mental healthcare system*" or "mental health care system*").ti,ab. (30745)

25 exp Needs Assessment/ (4457)

26 18 or 19 or 20 or 21 or 22 or 23 or 24 (707454)

27 25 and 26 (1902)

28 26 or 27 (707454)

29 12 and 17 and 28 (6782)

30 exp Treatment Barriers/ or ((implement* or innovat* or integrat* or uptake* or up-tak* or accept* or "scal* up" or scal*-up or up-scal* or adher* or promot* or adopt* or engag* or improvement* or intervention*) and (barrier* or impede or impediment* or facilitat* or challeng* or solution* or driver* or optimi* or diffus* or infus* or success* or limitation* or strateg* or approach* or hinder* or obstacle* or hurdle* or opportunit* or enabl* or sustain* or operational* or adapt* or capacit* or polic*)).ti,ab. (783039)

31 29 and 30 (3287)

32 limit 31 to yr="2010 -Current" (2940)

33 limit 32 to (chapter or "column/opinion" or "comment/reply" or dissertation or editorial or letter) (586)

34 32 not 33 (2354)

35 limit 34 to english language (2180)

***************************

**Scopus**

Results: 1169

INDEXTERMS ( *"telemedicine"* )  OR  INDEXTERMS ( *"telerehabilitation"* )  OR  ( TITLE-ABS ( *"telemedicine"* )  OR  TITLE-ABS ( *"tele-medicine"* )  OR  TITLE-ABS ( *"telemental"* )  OR  TITLE-ABS ( *"tele-mental"* )  OR  TITLE-ABS ( *"telehealth"* )  OR  TITLE-ABS ( *"tele-health"* )  OR  TITLE-ABS ( *"telepsychiatr*"* )  OR  TITLE-ABS ( *"tele-psychiatr*"* ) )  OR  INDEXTERMS ( *"biomedical technology"* )  OR  TITLE-ABS ( *"health technolog*"* )  OR  TITLE-ABS ( *"digital"* )  W/2  ( TITLE-ABS ( *"health"* )  OR  TITLE-ABS ( *"technolog*"* )  OR  TITLE-ABS ( *"revolution"* )  OR  TITLE-ABS ( *"intervention*"* ) )  OR  TITLE-ABS ( *"emerging technolog*"* )  OR  TITLE-ABS ( *"ehealth"* )  OR  TITLE-ABS ( *"e-health"* )  OR  TITLE-ABS ( *"e-mental"* )  OR  TITLE-ABS ( *"e-therap* electronic health"* )  OR  TITLE-ABS ( *"mhealth"* )  OR  TITLE-ABS ( *"m-health"* )  OR  TITLE-ABS ( *"mobile health"* )  OR  INDEXTERMS ( *"mobile applications"* )  OR  ( TITLE-ABS ( *"app"* )  OR  TITLE-ABS ( *"apps"* )  OR  TITLE-ABS ( *"app-based"* )  OR  TITLE-ABS ( *"appbased"* ) )  OR  ( TITLE-ABS ( *"mobile"* )  W/2  TITLE-ABS ( *"application*"* ) )  OR  INDEXTERMS ( *"internet"* )  OR  INDEXTERMS ( *"internet-based application"* )  OR  ( TITLE-ABS ( *"website*"* )  OR  TITLE-ABS ( *"web-site*"* )  OR  TITLE-ABS ( *"web-based"* )  OR  TITLE-ABS ( *"internet"* )  OR  TITLE-ABS ( *"internet-based"* )  OR  TITLE-ABS ( *"online"* )  OR  TITLE-ABS ( *"on-line"* ) )  AND  INDEXTERMS ( *"mental health"* )  OR  INDEXTERMS ( *"mental disorders"* )  OR  ( TITLE-ABS ( *"mental health"* )  OR  TITLE-ABS ( *"mental healthcare"* )  OR  TITLE-ABS ( *"behavio?ral health"* )  OR  TITLE-ABS ( *"mental disorder*"* )  OR  TITLE-ABS ( *"mental illness*"* ) )  OR  INDEXTERMS ( *"mental health services"* )  OR  INDEXTERMS ( *"community mental health services"* )  AND  INDEXTERMS ( *"delivery of health care"* )  OR  INDEXTERMS ( *"health care reform"* )  OR  INDEXTERMS ( *"health services accessibility"* )  OR  ( TITLE-ABS ( *"reform"* )  OR  TITLE-ABS ( *"reforms"* )  OR  TITLE-ABS ( *"reforming"* ) )  OR  INDEXTERMS ( *"Health Policy"* )  OR  INDEXTERMS ( *"economics"* )  OR  INDEXTERMS ( *"cost and cost analysis"* )  OR  TITLE-ABS ( *"funded"* )  OR  TITLE-ABS ( *"funding"* )  OR  TITLE-ABS ( *"underfund*"* )  OR  TITLE-ABS ( *"fragment*"* )  OR  TITLE-ABS ( *"welfare"* )  OR  ( TITLE-ABS ( *"healthcare system*"* )  OR  TITLE-ABS ( *"health care system*"* )  OR  TITLE-ABS ( *"health system*"* )  OR  TITLE-ABS ( *"mental healthcare system*"* )  OR  TITLE-ABS ( *"mental health care system*"* ) )  AND  ( ( TITLE-ABS ( *"implement*"* )  OR  TITLE-ABS ( *"innovat*"* )  OR  TITLE-ABS ( *"integrat*"* )  OR  TITLE-ABS ( *"uptake*"* )  OR  TITLE-ABS ( *"up-tak*"* )  OR  TITLE-ABS ( *"accept*"* )  OR  TITLE-ABS ( *"scal* up"* )  OR  TITLE-ABS ( *"scal*-up"* )  OR  TITLE-ABS ( *"up-scal*"* )  OR  TITLE-ABS ( *"adher*"* )  OR  TITLE-ABS ( *"promot*"* )  OR  TITLE-ABS ( *"adopt*"* )  OR  TITLE-ABS ( *"engag*"* )  OR  TITLE-ABS ( *"improvement*"* )  OR  TITLE-ABS ( *"intervention*"* ) )  AND  ( TITLE-ABS ( *"barrier*"* )  OR  TITLE-ABS ( *"impede"* )  OR  TITLE-ABS ( *"impediment*"* )  OR  TITLE-ABS ( *"facilitat*"* )  OR  TITLE-ABS ( *"challeng*"* )  OR  TITLE-ABS ( *"solution*"* )  OR  TITLE-ABS ( *"driver*"* )  OR  TITLE-ABS ( *"optimi*"* )  OR  TITLE-ABS ( *"diffus*"* )  OR  TITLE-ABS ( *"infus*"* )  OR  TITLE-ABS ( *"success*"* )  OR  TITLE-ABS ( *"limitation*"* )  OR  TITLE-ABS ( *"strateg*"* )  OR  TITLE-ABS ( *"approach*"* )  OR  TITLE-ABS ( *"hinder*"* )  OR  TITLE-ABS ( *"obstacle*"* )  OR  TITLE-ABS ( *"hurdle*"* )  OR  TITLE-ABS ( *"opportunit*"* )  OR  TITLE-ABS ( *"enabl*"* )  OR  TITLE-ABS ( *"sustain*"* )  OR  TITLE-ABS ( *"operational*"* )  OR  TITLE-ABS ( *"adapt*"* )  OR  TITLE-ABS ( *"capacit*"* )  OR  TITLE-ABS ( *"polic*"* ) ) )  AND  ( LIMIT-TO ( PUBYEAR ,  *2022* )  OR  LIMIT-TO ( PUBYEAR ,  *2021* )  OR  LIMIT-TO ( PUBYEAR ,  *2021* )  OR  LIMIT-TO ( PUBYEAR ,  *2020* )  OR  LIMIT-TO ( PUBYEAR ,  *2019* )  OR  LIMIT-TO ( PUBYEAR ,  *2018* )  OR  LIMIT-TO ( PUBYEAR ,  *2017* )  OR  LIMIT-TO ( PUBYEAR ,  *2016* )  OR  LIMIT-TO ( PUBYEAR ,  *2015* )  OR  LIMIT-TO ( PUBYEAR ,  *2014* )  OR  LIMIT-TO ( PUBYEAR ,  *2013* )  OR  LIMIT-TO ( PUBYEAR ,  *2012* )  OR  LIMIT-TO ( PUBYEAR ,  *2011* )  OR  LIMIT-TO ( PUBYEAR ,  *2010* ) )  AND  ( LIMIT-TO ( LANGUAGE ,  *"English"* ) )  AND  ( EXCLUDE ( DOCTYPE ,  *"cp"* )  OR  EXCLUDE ( DOCTYPE ,  *"no"* )  OR  EXCLUDE ( DOCTYPE ,  *"ed"* )  OR  EXCLUDE ( DOCTYPE ,  *"le"* ) )

*****************

**Web of Science**

Results 2494

#9 AND #8 AND #7 AND #6

Refined by: PUBLICATION YEARS: ( 2022 OR 2021 OR 2012 OR 2020 OR 2011 OR 2019 OR 2010 OR 2018 OR 2017 OR 2016 OR 2015 OR 2014 ) AND [excluding] DOCUMENT TYPES: ( PROCEEDINGS PAPER OR LETTER OR MEETING ABSTRACT OR EDITORIAL MATERIAL ) AND LANGUAGES: ( ENGLISH )

Indexes=SCI-EXPANDED, SSCI, A&HCI, CPCI-S, CPCI-SSH, ESCI, CCR-EXPANDED, IC Timespan=All years

#9 AND #8 AND #7 AND #6

Refined by: PUBLICATION YEARS: (2022 OR 2021 OR 2012 OR 2020 OR 2011 OR 2019 OR 2010 OR 2018 OR 2017 OR 2016 OR 2015 OR 2014 ) AND [excluding] DOCUMENT TYPES: ( PROCEEDINGS PAPER OR LETTER OR MEETING ABSTRACT OR EDITORIAL MATERIAL )

Indexes=SCI-EXPANDED, SSCI, A&HCI, CPCI-S, CPCI-SSH, ESCI, CCR-EXPANDED, IC Timespan=All years

#9 AND #8 AND #7 AND #6

Refined by: PUBLICATION YEARS: (2022 OR 2021 OR 2012 OR 2020 OR 2011 OR 2019 OR 2010 OR 2018 OR 2017 OR 2016 OR 2015 OR 2014 )

Indexes=SCI-EXPANDED, SSCI, A&HCI, CPCI-S, CPCI-SSH, ESCI, CCR-EXPANDED, IC Timespan=All years

#9 AND #8 AND #7 AND #6

Indexes=SCI-EXPANDED, SSCI, A&HCI, CPCI-S, CPCI-SSH, ESCI, CCR-EXPANDED, IC Timespan=All years

TS=((implement* or innovat* or integrat* or uptake or up-take or accept* or "scale up" or scale-up or adher* or promot* or adopt* or engag* or improvement* or intervention*) AND (barrier* or impede or impediment* or facilitat* or challenge* or solution* or driver* or optimi* or diffus* or infus* or success* or limitation* or strateg* or approach* or hinder* or obstacle* or hurdle* or opportunit* or enabl* or sustain* or operational* or adapt* or capacit* or polic*) )

Indexes=SCI-EXPANDED, SSCI, A&HCI, CPCI-S, CPCI-SSH, ESCI, CCR-EXPANDED, IC Timespan=All years

TS=("health care reform*" or "healthcare reform" or policy or policies or economic* or "health care system*" or "healthcare system*" or "health system*" or "mental heath care system*" or "mental heathcare system*" or cost* or "healthcare cost*" or expenditure* or "delivery of health care" or "delivery of healthcare" or "healthcare delivery" or "health care delivery" or "health service* research")

Indexes=SCI-EXPANDED, SSCI, A&HCI, CPCI-S, CPCI-SSH, ESCI, CCR-EXPANDED, IC Timespan=All years

TS=("mental health" or "mental disorder*" or "mental healthcare" or "mental illness*" or "behavioural health" or "behavioral health")

Indexes=SCI-EXPANDED, SSCI, A&HCI, CPCI-S, CPCI-SSH, ESCI, CCR-EXPANDED, IC Timespan=All years

#5 OR #4 OR #3 OR #2 OR #1

Indexes=SCI-EXPANDED, SSCI, A&HCI, CPCI-S, CPCI-SSH, ESCI, CCR-EXPANDED, IC Timespan=All years

TS=(internet or internet-based or website or web-site or web-based or internet-based or online or on-line)

Indexes=SCI-EXPANDED, SSCI, A&HCI, CPCI-S, CPCI-SSH, ESCI, CCR-EXPANDED, IC Timespan=All years

TS=("mobile app*" or app or apps or app-based or appbased)

Indexes=SCI-EXPANDED, SSCI, A&HCI, CPCI-S, CPCI-SSH, ESCI, CCR-EXPANDED, IC Timespan=All years

TS=(ehealth or e-health or e-mental or e-therap* or "electronic health" or mhealth or m-health or "mobile health")

Indexes=SCI-EXPANDED, SSCI, A&HCI, CPCI-S, CPCI-SSH, ESCI, CCR-EXPANDED, IC Timespan=All years

TS=("health technolog*" or "digital health" or "digital technolog*" or "digital revolution" or "digital intervention*"or "emerging technolog*")

Indexes=SCI-EXPANDED, SSCI, A&HCI, CPCI-S, CPCI-SSH, ESCI, CCR-EXPANDED, IC Timespan=All years

TS=(telemedicine or tele-medicine or telemental or tele-mental or telehealth or tele-health or telepsychiatr* or tele-psychiatr*)

Indexes=SCI-EXPANDED, SSCI, A&HCI, CPCI-S, CPCI-SSH, ESCI, CCR-EXPANDED, IC Timespan=All years

Table A3: Data items extracted from selected studies

| **Study Description** | **Findings** |
| --- | --- |
| Year | Digital Health Technology |
| Type of Study | Digital Technology feature (guided, unguided) |
| Methodology (qualitative, quantitative) | Context (nano, micro, meso, macro) |
| Method  Sample  Country (single, multiple) | Barriers  Facilitators |
| Study Setting (global, European, international) |  |
| Technology users |  |
| Mental Health Disorder |  |

Table A4: JBI critical appraisal checklist for qualitative research

| Reviewer_____________________________________________ Date_______________________________  Author_______________________________________ Year_________________ Record Number_________   \|  \| Yes \| No \| Unclear \| Not applicable \| \| --- \| --- \| --- \| --- \| --- \| \| 1. Is there congruity between the stated philosophical perspective and the research methodology? \| □ \| □ \| □ \| □ \| \| 1. Is there congruity between the research methodology and the research question or objectives? \| □ \| □ \| □ \| □ \| \| 1. Is there congruity between the research methodology and the methods used to collect data? \| □ \| □ \| □ \| □ \| \| 1. Is there congruity between the research methodology and the representation and analysis of data? \| □ \| □ \| □ \| □ \| \| 1. Is there congruity between the research methodology and the interpretation of results? \| □ \| □ \| □ \| □ \| \| 1. Is there a statement locating the researcher culturally or theoretically? \| □ \| □ \| □ \| □ \| \| 1. Is the influence of the researcher on the research, and vice- versa, addressed? \| □ \| □ \| □ \| □ \| \| 1. Are participants, and their voices, adequately represented? \| □ \| □ \| □ \| □ \| \| 1. Is the research ethical according to current criteria or, for recent studies, and is there evidence of ethical approval by an appropriate body? \| □ \| □ \| □ \| □ \| \| 1. Do the conclusions drawn in the research report flow from the analysis, or interpretation, of the data? \| □ \| □ \| □ \| □ \|   Overall appraisal: Include □ Exclude □ Seek further info □  Comments (Including reason for exclusion)  _________________________________________________________________________ |
| --- | --- | --- | --- | --- | --- | --- | --- | --- | --- | --- | --- | --- | --- | --- | --- | --- | --- | --- | --- | --- | --- | --- | --- | --- | --- | --- | --- | --- | --- | --- | --- | --- | --- | --- | --- | --- | --- | --- | --- | --- | --- | --- | --- | --- | --- | --- | --- | --- | --- | --- | --- | --- | --- | --- | --- |

Table A5: JBI critical appraisal of included studies

|  |  | **Category 1: Theoretical basis** | **Category 2: Method** | | | | **Category 3: Researcher influence** | | **Category 4: Participants** | | **Category 5: Interpretation of results** |
| --- | --- | --- | --- | --- | --- | --- | --- | --- | --- | --- | --- |
| **Author** | **Year** | **Q1** | **Q2** | **Q3** | **Q4** | **Q5** | **Q6** | **Q7** | **Q8** | **Q9** | **Q10** |
| Anastasiadou et al., (1) | 2019 | No | Yes | Yes | Yes | Yes | No | Yes | Yes | Yes | Yes |
| Bauer et al., (2) | 2018 | Yes | Yes | Yes | Yes | Yes | Yes | Yes | Yes | Yes | Yes |
| Bennett-Levy et al., (3) | 2017 | Yes | Yes | Yes | Yes | Yes | No | Yes | Yes | Yes | Yes |
| Berry et al., (4) | 2017 | No | Yes | Yes | Yes | Yes | Yes | Yes | Yes | Yes | Yes |
| Blease et al., (5) | 2020 | Yes | Yes | Yes | Yes | Yes | No | No | Yes | Yes | Yes |
| Bleyel et al., (6) | 2020 | Yes | Yes | Yes | Yes | Yes | No | Yes | Yes | Yes | Yes |
| Bruno et al., (7) | 2015 | Unclear | Unclear | Unclear | Yes | Yes | No | No | Yes | No | Unclear |
| Bucci et al., (8) | 2018 | Yes | Yes | Yes | Yes | Yes | No | Yes | Yes | No | Yes |
| Burchert et al., (9) | 2019 | Yes | Yes | Yes | Yes | Yes | No | Yes | Yes | Yes | Yes |
| Cárdenas et al., (10) | 2020 | No | Yes | Yes | Yes | Yes | No | Yes | Yes | Yes | Yes |
| Cheng et al., (11) | 2021 | Yes | Yes | Yes | Yes | Yes | No | Yes | Yes | Yes | Yes |
| Clarke et al., (12) | 2015 | Yes | Yes | Yes | Yes | Yes | No | Yes | Yes | Yes | Yes |
| Dederichs et al., (13) | 2021 | Unclear | Yes | Yes | Yes | Yes | No | Yes | Yes | Yes | Yes |
| Dingwal et al., (14) | 2015 | Unclear | Yes | Yes | Yes | Yes | Unclear | No | Yes | Yes | Yes |
| Dinkel et al., (15) | 2021 | Yes | Yes | Yes | Yes | Yes | No | No | Yes | Yes | Yes |
| Eccles et al., (16) | 2020 | No | Yes | Yes | Yes | Yes | No | Yes | Yes | Yes | Yes |
| Feijt et al., (17) | 2018 | Yes | Yes | Yes | Yes | Yes | No | No | Yes | Yes | Yes |
| Folker et al., (18) | 2018 | Unclear | Yes | Yes | Yes | Yes | No | No | Yes | Yes | Yes |
| Gaebel et al., (19) | 2020 | Yes | Yes | Yes | Yes | Yes | No | Yes | Yes | No | Yes |
| Gericke et al., (20) | 2021 | Unclear | Yes | Yes | Yes | Yes | No | No | Yes | Yes | Yes |
| Gordon et al., (21) | 2021 | Yes | Yes | Yes | Yes | Yes | No | No | Yes | Yes | Yes |
| Gould et al., (22) | 2019 | Unclear | Yes | Yes | Yes | Yes | No | No | Yes | Yes | Yes |
| Hadjistavr et al.,(23) | 2017 | Yes | Yes | Yes | Yes | Yes | No | No | Yes | Yes | Yes |
| Hawke et al., (24) | 2021 | No | Yes | Yes | Yes | Yes | Yes | No | Yes | Yes | Yes |
| Hermes et al., (25) | 2019 | Yes | Yes | Yes | Yes | Yes | Yes | Yes | Yes | Yes | Yes |
| Howland et al., (26) | 2021 | Yes | Yes | Yes | Yes | Yes | No | Unclear | Yes | No | Yes |
| Jonk et al., (27) | 2021 | Yes | Yes | Yes | Yes | Yes | No | Yes | Yes | Unclear | Yes |
| Kenicer et al., (28) | 2012 | Yes | Yes | Yes | Yes | Yes | No | No | No | No | Unclear |
| Kurki et al., (29) | 2018 | Yes | Yes | Yes | Yes | Yes | Yes | Yes | Yes | Yes | Yes |
| Lambert et al.,(30) | 2015 | Yes | Yes | Yes | Yes | Yes | No | No | No | Yes | Yes |
| La Monica et al., (31) | 2020 | Yes | Yes | Yes | Yes | Yes | No | No | No | Yes | Yes |
| Landes et al., (32) | 2021 | No | Yes | Yes | Yes | Yes | Yes | No | Yes | Unclear | Yes |
| Lattie et al., (33) | 2019 | Yes | Yes | Yes | Yes | Yes | No | Yes | Yes | Yes | Yes |
| Lord et al., (34) | 2016 | Yes | Yes | Yes | Yes | Yes | No | Yes | Yes | Yes | Yes |
| Lorenz et al., (35) | 2021 | No | Yes | Yes | Yes | Yes | No | No | Yes | Yes | Yes |
| Magal et al., (36) | 2021 | No | Yes | Yes | Yes | Yes | Yes | No | Yes | Yes | Yes |
| Mar M et al., (37) | 2014 | No | Yes | Yes | Yes | Yes | No | Yes | Yes | Yes | Yes |
| Margolis et al., (38) | 2018 | Yes | Yes | Yes | Yes | Yes | No | Yes | No | No | Yes |
| Matanow et al., (39) | 2021 | No | Yes | Yes | Yes | Yes | Yes | No | Yes | Yes | Yes |
| Matthews et al., (40) | 2017 | Yes | Yes | Yes | Yes | Yes | No | Yes | Yes | Yes | Yes |
| Meisel et al., (41) | 2018 | Yes | Yes | Yes | Yes | Yes | No | Yes | Yes | Yes | Yes |
| Melcher et al., (42) | 2020 | Yes | Yes | Yes | Unclear | Yes | No | No | Yes | Yes | Unclear |
| Melia et al., (43) | 2021 | Yes | Yes | Yes | Yes | Yes | No | No | Yes | Yes | Yes |
| Mercado et al., (44) | 2021 | No | Yes | Yes | Yes | Yes | No | No | Yes | Yes | Yes |
| Morgieve et al., (45) | 2019 | Yes | Yes | Yes | Yes | Yes | No | Yes | Yes | Yes | Yes |
| Newman et al., (46) | 2016 | Yes | Yes | Yes | Yes | Yes | No | No | Yes | No | Yes |
| Orlowski et al., (47) | 2016 | Yes | Yes | Yes | Yes | Yes | No | No | Unclear | Yes | Yes |
| Perry K et al., (48) | 2020 | Yes | Yes | Yes | Yes | Yes | No | No | Yes | yes | Yes |
| Pithara et al., (49) | 2020 | Yes | Yes | Yes | Yes | Yes | No | No | Yes | Yes | Yes |
| Pokhrel et al., (50) | 2021 | Yes | Yes | Yes | Yes | Yes | No | No | Yes | Yes | Yes |
| Pung et al., (51) | 2018 | Yes | Yes | Yes | Yes | Yes | No | No | Yes | Yes | Yes |
| Puszka et al., (52) | 2016 | Yes | Yes | Yes | Yes | Yes | No | Yes | Yes | Yes | Unclear |
| Pywell et al., (53) | 2020 | No | Yes | Yes | Yes | Yes | No | No | Yes | Yes | Yes |
| Raphiphatthana et al., (implementation)(54) | 2020 | Yes | Yes | Yes | Yes | Yes | No | No | Yes | Yes | Yes |
| Raphiphatthana et al., (interview)(55) | 2020 | Yes | Yes | Yes | Yes | Yes | No | Yes | Yes | No | Yes |
| Reger et al., (56) | 2017 | Yes | Yes | Yes | Yes | Yes | Yes | No | Yes | No | Yes |
| Renfrew et al., (57) | 2021 | Yes | Yes | Yes | Yes | Yes | No | Yes | Yes | Yes | Yes |
| Richards et al., (58) | 2018 | No | Yes | Yes | Yes | Yes | Yes | No | Yes | Yes | Yes |
| Rodda et al., (59) | 2019 | Yes | Yes | Yes | Yes | Yes | No | No | Yes | Yes | Yes |
| Rozental et al., (60) | 2020 | Yes | Yes | Yes | Yes | Yes | No | Yes | Yes | Yes | Yes |
| Rushton et al., (61) | 2019 | Yes | Yes | Yes | Yes | Yes | Yes | Yes | Yes | Yes | Yes |
| Rushton et al., (62) | 2020 | Yes | Yes | Yes | Yes | Yes | No | Yes | Yes | Yes | Yes |
| Schneider et al., (63) | 2014 | Unclear | Yes | Yes | Yes | Yes | No | No | Yes | Yes | Yes |
| Shealy et al., (64) | 2015 | Yes | Yes | Yes | Yes | Yes | No | No | Unclear | Unclear | Yes |
| Simms et al., (65) | 2011 | Yes | Yes | Yes | Yes | Yes | No | No | Yes | Yes | Yes |
| Sinclair et al., (66) | 2013 | Yes | Yes | Yes | Yes | Yes | Yes | No | Yes | Yes | Yes |
| Skime et al., (67) | 2021 | No | Yes | Yes | Yes | Yes | No | No | Yes | Unclear | Yes |
| Sogomonjan et al., (68) | 2019 | No | Yes | Yes | Yes | Yes | No | No | Yes | No | Yes |
| Steare et al., (69) | 2021 | No | Yes | Yes | Yes | Yes | Yes | Yes | Yes | Yes | Yes |
| Stjernsward et al., (70) | 2017 | Yes | Yes | Yes | Yes | Yes | No | No | Yes | Yes | Yes |
| Sturk et al., (71) | 2019 | No | Yes | Yes | Yes | Yes | No | No | Yes | Yes | Yes |
| Taimen et al., (72) | 2018 | Yes | Yes | Yes | Yes | Yes | No | No | Yes | No | Yes |
| Tobbit et al., (73) | 2019 | Yes | Yes | Yes | Yes | Yes | No | No | No | Yes | Unclear |
| Tonnies et al., (74) | 2021 | Yes | Yes | Yes | Yes | Yes | Yes | Yes | Yes | Yes | Yes |
| Town et al., (75) | 2017 | Yes | Yes | Yes | Yes | Yes | No | No | Yes | Yes | Yes |
| Traube et al., (76) | 2021 | Yes | Yes | Yes | Yes | Yes | Yes | Yes | Yes | Yes | Yes |
| Uscher Pines et al., (77) | 2020 | No | Yes | Yes | Yes | Yes | No | No | Unclear | Yes | Yes |
| Volpe et al., (78) | 2014 | Yes | Yes | Yes | Yes | Yes | Yes | Yes | Yes | No | Yes |
| Wozney et al., (79) | 2017 | Yes | Yes | Yes | Yes | Yes | No | Yes | Yes | Yes | Yes |
| Wynn et al., (80) | 2012 | Yes | Yes | Yes | No | Yes | No | No | No | No | No |
| Ye et al., (81) | 2021 | Yes | Yes | Yes | Yes | Yes | No | No | Yes | Yes | Yes |
| **Qualitative domains:**  **Category 1**: Theoretical framework - High quality (green) = 1 yes; Medium quality (yellow)= unclear; Low quality (red)= 0 yes.  **Category 2**: Design and methodology - High quality (green) = 3 or more yeses; Medium quality (orange) = 2 yeses; Low quality (red) = 2 or less yeses.  **Category 3**: Researcher influence - High quality (green) = 2 yeses; Medium quality (orange) = 1 yes; Low quality (red) = 0 yeses.  **Category 4**: Participants - High quality (green) = 2 yeses; Medium quality (orange) = 1 yes; Low quality (red) = 0 yeses.  **Category 5**: Interpretation of results - High quality (green) = yes; Medium quality (yellow)= unclear; Low quality (red)= 0 yes | | | | | | | | | | | |

Table A6: ENTREQ Checklist

| **No** | **Item** | **Guide and description** | **Location where item is reported** |
| --- | --- | --- | --- |
| **1** | Aim | State the research question the synthesis addresses. | Page 1 |
| **2** | Synthesis methodology | Identify the synthesis methodology or theoretical framework which underpins the synthesis, and describe the rationale for choice of methodology *(e.g. meta-ethnography, thematic synthesis, critical interpretive synthesis, grounded theory synthesis, realist synthesis, meta-aggregation, meta-study, framework synthesis).* | Page 3 |
| **3** | Approach to searching | Indicate whether the search was pre-planned (*comprehensive search strategies to seek all available studies)* or iterative (*to seek all available concepts until they theoretical saturation is achieved)*. | Page 2 |
| **4** | Inclusion criteria | Specify the inclusion/exclusion criteria *(e.g. in terms of population, language, year limits, type of publication, study type).* | Page 2 |
| **5** | Data sources | Describe the information sources used (e.g. *electronic databases (MEDLINE, EMBASE, CINAHL, psycINFO, Econlit), grey literature databases (digital thesis, policy reports), relevant organisational websites, experts, information specialists, generic web searches (Google Scholar) hand searching, reference lists)* and when the searches conducted; provide the rationale for using the data sources. | Page 2 |
| **6** | Electronic Search strategy | Describe the literature search *(e.g. provide electronic search strategies with population terms, clinical or health topic terms, experiential or social phenomena related terms, filters for qualitative research, and search limits)*. | Appendix Table 3 |
| **7** | Study screening methods | Describe the process of study screening and sifting *(e.g. title, abstract and full text review, number of independent reviewers who screened studies).* | Page 3 |
| **8** | Study characteristics | Present the characteristics of the included studies *(e.g. year of publication, country, population, number of participants, data collection, methodology, analysis, research questions).* | Appendix Table 8 |
| **9** | Study selection results | Identify the number of studies screened and provide reasons for study exclusion *(e,g, for comprehensive searching, provide numbers of studies screened and reasons for exclusion indicated in a figure/flowchart; for iterative searching describe reasons for study exclusion and inclusion based on modifications t the research question and/or contribution to theory development).* | Figure 1 |
| **10** | Rationale for appraisal | Describe the rationale and approach used to appraise the included studies or selected findings *(e.g. assessment of conduct (validity and robustness), assessment of reporting (transparency), assessment of content and utility of the findings).* | Appendix Table 6, 10 |
| **11** | Appraisal items | State the tools, frameworks and criteria used to appraise the studies or selected findings *(e.g. Existing tools: CASP, QARI, COREQ, Mays and Pope* [[25](https://bmcmedresmethodol.biomedcentral.com/articles/10.1186/1471-2288-12-181#ref-CR25)]*; reviewer developed tools; describe the domains assessed: research team, study design, data analysis and interpretations, reporting).* | Page 6 |
| **12** | Appraisal process | Indicate whether the appraisal was conducted independently by more than one reviewer and if consensus was required. | Page 6 |
| **13** | Appraisal results | Present results of the quality assessment and indicate which articles, if any, were weighted/excluded based on the assessment and give the rationale. | Table 6 |
| **14** | Data extraction | Indicate which sections of the primary studies were analysed and how were the data extracted from the primary studies? *(e.g. all text under the headings “results /conclusions” were extracted electronically and entered into a computer software).* | Page 5 |
| **15** | Software | State the computer software used, if any. | Page 3 |
| **16** | Number of reviewers | Identify who was involved in coding and analysis. | Page 3 |
| **17** | Coding | Describe the process for coding of data *(e.g. line by line coding to search for concepts).* | Page 3 |
| **18** | Study comparison | Describe how were comparisons made within and across studies *(e.g. subsequent studies were coded into pre-existing concepts, and new concepts were created when deemed necessary).* | Page 3 |
| **19** | Derivation of themes | Explain whether the process of deriving the themes or constructs was inductive or deductive. | Page 3 |
| **20** | Quotations | Provide quotations from the primary studies to illustrate themes/constructs, and identify whether the quotations were participant quotations of the author’s interpretation. | Appendix Table 9 |
| **21** | Synthesis output | Present rich, compelling and useful results that go beyond a summary of the primary studies (e.g. *new interpretation, models of evidence, conceptual models, analytical framework, development of a new theory or construct).* | Table 1 |

Table A7: Studies description

| **Author** | **Year** | **Type of study** | **Methodology** | **Method** | **Sample**  **size** | **Country** | **Study setting** | **Technology users/ Population** | **Mental Health Disorder** | **Digital Technologies** | **Technology feature** |
| --- | --- | --- | --- | --- | --- | --- | --- | --- | --- | --- | --- |
| Anastasiadou et al., (1) | 2019 | Focus Group | Qualitative | Open-ended questions | 38  (11 experts,  10 healthcare professionals,  9 patients,  8 ED specialists) | Spain | Local | General population | Eating disorder | Mobile Health | Guided |
| Bauer et al., (2) | 2018 | Evaluation | Mixed methods | Semi-structured Interviews | 16 providers | United States | Local | General population | Bipolar Disorder | Telehealth | Guided |
| Bennett-Levy et al., (3) | 2017 | Evaluation | Qualitative | Semi-structured Interviews | 50 providers of services for Aboriginal and Torres Strait Islander peoples | Australia | Local | General population | General Mental Health | General Digital Health | Guided |
| Berry et al., (4) | 2017 | Focus Group | Qualitative | Topic guide | 20 staff working in mental health care services  12 secondary care psychological services  4 rehabilitation unit  4  community mental health team | UK | Local | General population | Severe mental health disorders | Mobile Health | Guided |
| Blease et al., (5) | 2020 | Survey | Mixed methods | Open-ended questions | 791 psychiatrists | France Italy Germany UK Russian Federation Spain Australia Belgium Netherlands Japan Mexico Canada Greece China Brazil Turkey Switzerland Norway Portugal India | Global | General population | General Mental Health | Artificial Intelligence | Guided |
| Bleyel et al., (6) | 2020 | Evaluation | Qualitative | Semi-structured Interviews | 13 patients from primary care practices and a tertiary  care hospital | Germany | Local | General population | General Mental Health | Video consultation | Guided |
| Bruno et al., (7) | 2015 | Survey | Mixed methods | Not specified | 86 health professionals | Australia | Local | General population | General Mental Health | Internet of things | Guided |
| Bucci et al., (8) | 2018 | Framework analysis | Qualitative | Semi-structured Interviews | 21 people registered  with early intervention for psychosis services | UK | Local | General population | Psychosis | Mobile Health | Guided |
| Burchert et al., (9) | 2019 | Focus Group | Qualitative | Free list interviews | 128 adult Syrian refugees residing in Germany, Sweden  and Egypt | Germany Sweden Egypt | Global | Syrian Refugee | General Mental Health | Mobile Health | Guided |
| Cárdenas et al., (10) | 2020 | Focus Group | Qualitative | Semi-structured Interviews | 16 focus groups: health professionals,  administrative professionals, patients, and community organization representatives | Colombia | Local | General population | Depression | General Digital Health | Guided |
| Cheng et al., (11) | 2021 | Workshop | Qualitative | Not specified | 105 participants:  75 clients  7 support members  21 staff members  2 (missing) | Australia | Local | Young people | General Mental Health | Platform | Guided |
| Clarke et al., (12) | 2015 | Survey | Mixed methods | Semi-structured Interviews | 67 chief information officers | UK | Local | General population | General Mental Health | Electronic record system | Guided |
| Dederichs et al., (13) | 2021 | Case Study | Qualitative | Not specified | 26 medical students | Germany | Local | Medical students | General Mental Health | Apps | Unguided |
| Dingwal et al., (14) | 2015 | Expert Group | Qualitative | Semi-structured Interviews | 15 service providers and managers from a range of rural and remote primary health care service | Australia | Local | General population | General Mental Health | Apps | Guided |
| Dinkel et al., (15) | 2021 | Case Study | Qualitative | Semi-structured Interviews | 32 participants:  17 patients, 15 healthcare providers and staff | United States | Local | General population | General Mental Health | Apps | Guided |
| Eccles et al., (16) | 2020 | Interview | Qualitative | Semi-structured Interviews | 77 participants at high risk for  depression | Canada | Local | Men | Depression | Web Based program/platform | Guided |
| Feijt et al., (17) | 2018 | Interview | Qualitative | Semi-structured Interviews | 12 clinical  psychologists | Netherlands | Local | General population | General Mental Health | General Digital Health | Guided |
| Folker et al., (18) | 2018 | Interview | Qualitative | Semi-structured Interviews | 21 participants  9 management of the service  15 Key staff | Sweden Netherlands Norway Denmark  Scotland | European | General population | General Mental Health | Computerised CBT | Guided |
| Gaebel et al., (19) | 2020 | Stakeholders interviews | Qualitative | Semi-structured Interviews | 52 participants  11 health professionals  4 service providers  2 Individuals who have experienced  mental health problems  3 Patients associations/organizations  9 healthcare policymakers  5 researchers  11 small and medium enterprises  7 others | France Germany Belgium Ireland UK Netherlands | European | General population | General Mental Health | General Digital Health | Not specified |
| Gericke et al., (20) | 2021 | Interview | Qualitative | Semi-structured Interviews | 9 students with | South Africa | Local | University Students | Moderate to moderately severe  symptoms of depression | Computerised CBT | Guided |
| Gordon et al., (21) | 2021 | Interview | Mixed methods | Not specified | 542 participants (program user) | Canada | Local | General population | General Mental Health | Web Based program/platform | Unguided |
| Gould et al., (22) | 2019 | Interview | Mixed methods | Semi-structured Interviews | 77 Veterans | United States | Local | Veterans | General Mental Health | General Digital Health | Not specified |
| Hadjistavr et al.,(23) | 2017 | Survey | Qualitative | Open-ended questions | 33 participants  22 therapists  11 managers | Canada | Local | General population | General Mental Health | Platform | Guided |
| Hawke et al., (24) | 2021 | Survey | Mixed methods | Not specified | 409 youth | Canada | Local | Young people | General Mental Health | General Digital Health | Not specified |
| Hermes et al.,(25) | 2019 | Interview | Qualitative | Semi-structured Interviews | 20 participants  12 providers, 8 administrators and policy makers | United States | Local | Veterans | General Mental Health | General Digital Health | Unguided |
| Howland et al., (26) | 2021 | Focus Group | Qualitative | Semi-structured Interviews | 14 participants:  10 tele psychiatrists  4 telepsychologygistd | United States | Local | General population | General Mental Health | Telehealth | Guided |
| Jonk et al., (27) | 2021 | Interview | Mixed methods | Semi-structured Interviews | 16 administrative and clinical leaders | United States | Local | General population | General Mental Health | Telehealth | Guided |
| Kenicer et al., (28) | 2012 | Survey | Qualitative | Structured interviews | 14 IT managers/IT security  managers | UK | Local | General population | General Mental Health | Computerised CBT | Guided |
| Kurki et al., (29) | 2018 | Interview | Qualitative | Semi-structured Interviews | 9 nurses at  two psychiatric outpatient clinic | Finland | Local | Adolescents | General Mental Health | Internet based support system | Guided |
| Lambert et al.,(30) | 2015 | Expert Group | Qualitative | Semi-structured Interviews | 53 participants from programs administrators | United States | Local | General population | General Mental Health | Telehealth | Guided |
| La Monica et al., (31) | 2020 | Survey | Mixed methods | Semi-structured Interviews | 47 participants  12 General psychologist,  10 Social worker,  6 Counselor,  4 Service managers and administrators, 2 Mental health nurse, 2 Youth worker, 1 Dentist, 1 General practitioner, 9 Other | Australia | Local | General population | General Mental Health | Platform | Guided |
| Landes et al., (32) | 2021 | Survey | Mixed methods | Adaptive questions | 32 providers | United States | Local | Veterans | Borderline Personality Disorder | Telehealth | Guided |
| Lattie et al., (33) | 2019 | Focus Group | Qualitative | Not specified | 57 staff members | United States | Local | General population | General Mental Health Suicidality | Internet of things | Guided |
| Lord et al., (34) | 2016 | Stakeholders interviews | Qualitative | Not specified | 12 leadership and provider stakeholders | United States | Local | Veterans | Substance use | Apps | Not specified |
| Lorenz et al., (35) | 2021 | Interview | Qualitative | Semi-structured Interviews | 29 clients, social network members, and professionals of  an ambulatory team | Netherlands | Local | General population | General Mental Health | General Digital Health | Not specified |
| Magal et al., (36) | 2021 | Case Study | Qualitative | In depth interview | 27 psychiatrists | Israel | Local | General population | General Mental Health | Telehealth | Guided |
| Mar M et al., (37) | 2014 | Survey | Qualitative | Themed interviews | 23 Generation Y participants | Canada | Local | Generation Y | Depression | Internet of things | Guided |
| Margolis et al., (38) | 2018 | Survey | Mixed methods | Not specified | 29 patients  16 multidisciplinary medical team | United States | Local | Children | Depression | Telehealth | Guided |
| Matanow et al., (39) | 2021 | Interview | Qualitative | Semi-structured Interviews | 16 mental health professionals and 29 service users | UK | Local | General population | Depression | Apps | Guided |
| Matthews et al., (40) | 2017 | Interview | Qualitative | Semi-structured Interviews | 37 providers | United States | Local | General population | General Mental Health | Electronic record system | Guided |
| Meisel et al., (41) | 2018 | Survey | Qualitative | Open-ended questions | 33 therapists  11 PWPs  8 clinical psychologists  7 high intensity CBT therapist  3 counsellors  2 psychologists  2 assistant psychologists | UK | Local | General population | General Mental Health | Computerised CBT | Guided |
| Melcher et al.,(42) | 2020 | Interview | Mixed methods | Not specified | 100 college students | United States | Local | College students | General Mental Health | Apps | Not specified |
| Melia et al., (43) | 2021 | Interview | Qualitative | Semi-structured Interviews | 15 mental health professionals and clinician managers | Ireland | Local | General population | General Mental Health | Apps | Guided |
| Mercado et al., (44) | 2021 | Interview | Qualitative | Semi-structured Interviews | 11 behavioural health professionals | United States | Local | General population | General Mental Health | Thelephone | Guided |
| Morgieve et al., (45) | 2019 | Focus Group | Qualitative | Semidirective interview | 70 participants  5 general practitioners, 5 psychiatrists, 7 psychologists5 social workers,  9 occupational therapists,  9 nurses,  12 services users,  3 user representatives,  6 general  public  9 family caregivers | France | Local | General population | General Mental Health | General Digital Health | Not specified |
| Newman et al., (46) | 2016 | Interview | Qualitative | Open-ended questions | 40 participants, managers,  team leaders, senior clinicians, mental health nurse practitioners  and administrative staff | Australia | Local | General population | General Mental Health | Telehealth | Guided |
| Orlowski et al., (47) | 2016 | Focus Group | Qualitative | Semi-structured Interviews | 48 general and mental health  youth workers | Australia | Local | Young people | General Mental Health | General Digital Health | Not specified |
| Perry K et al., (48) | 2020 | Survey | Qualitative | Not specified | 159 mental health staff | United States | Local | General population | General Mental Health | Telehealth | Guided |
| Pithara et al., (49) | 2020 | Interview | Qualitative | Not specified | 20 providers  15 (mental health support workers, peer support  workers, psychiatrists, occupational therapists, community  psychiatric nurses, and social workers  5 managers | UK | Local | General population | General Mental Health | Specific technology | Guided |
| Pokhrel et al., (50) | 2021 | Focus Group | Qualitative | Semi-structured Interviews | 43 participants  8 medical officers  35 primary healthcare workers | Nepal | Local | General population | General Mental Health | App | Guided |
| Pung et al., (51) | 2018 | Interview | Qualitative | Semi-structured Interviews | 16 patients | Australia | Local | General population | Depression | Apps | Not specified |
| Puszka et al., (52) | 2016 | Interview | Qualitative | Semi-structured Interviews | 32  21 managers, directors, chief executive officers (CEOs), 11 senior  practitioners | Australia | Local | Aboriginal and Torres Strait Islander Australians | General Mental Health | General Digital Health | Not specified |
| Pywell et al., (53) | 2020 | Interview | Qualitative | Semi-structured Interviews | 10 older adults | UK | Local | Older adults | General Mental Health | Mobile Health | Not specified |
| Raphiphatthana et al., (54) | 2020 | Survey | Mixed methods | Open-ended questions | 65 participants:  3 Indigenous Health Worker  1 Aboriginal Mental Health Worker  6 Aboriginal Community Worker  4 Alcohol and Other Drug Worker  13 Nurse  2 Psychologist 1 GP 9 Social Worker  2 Occupational Therapist  3 Trainer/Educator  6 Manager/Coordinator/CEO | Australia | Local | Aboriginal and Torres Strait Islander Australians | General Mental Health | General Digital Health | Not specified |
| Raphiphatthana et al., (55) | 2020 | Interview | Qualitative | Semi-structured Interviews | 57 service providers working with Aboriginal and Torres Strait Islander  people | Australia | Local | Aboriginal and Torres Strait Islander Australians | General Mental Health | General Digital Health | Guided |
| Reger et al., (56) | 2017 | Interview | Qualitative | Semi-structured Interviews | 25 providers | United States | Local | Veterans | PTSD | Apps | Guided |
| Renfrew et al.,(57) | 2021 | Interview | Qualitative | Not specified | 320 potential users  Seventh-day Adventist Church members | Australia New Zeland | Local | General population | General Mental Health | Text messages | Guided |
| Richards et al.,(58) | 2018 | Focus Group | Mixed methods | Semi-structured Interviews | 13 participants:  6 psychologists  7 patients | Australia | Local | General population | General Mental Health | Web Based program/platform | Guided |
| Rodda et al., (59) | 2019 | Interview | Qualitative | Semi-structured Interviews | 7 therapists | Australia | Local | General population | Gambling | Computerised CBT | Guided |
| Rozental et al., (60) | 2020 | Interview | Qualitative | Open-ended questions | 30 clients | UK | Local | General population | Perfectionism | Computerised CBT | Guided |
| Rushton et al., (62) | 2019 | Focus Group | Qualitative | Semi-structured Interviews | 21 decision makers | UK | Local | General population | Depression | Thelephone | Guided |
| Rushton et al.,(61) | 2020 | Interview | Qualitative | Semi-structured Interviews | 28 patients | UK | Local | General population | General Mental Health | Thelephone | Guided |
| Schneider et al., (63) | 2014 | Interview | Mixed methods | Open-ended questions | 359 patients | UK | Local | General population | Depression | General Digital Health | Unguided |
| Shealy et al., (64) | 2015 | Interview/ Case study | Qualitative | Semi Structured Interviews | 2 participants  1 patient  1 caregiver | United States | Local | Adolescents | Depression | Telehealth |  |
| Simms et al., (65) | 2011 | Interview | Mixed methods | Semi Structured Interviews | 25 participants  4 psychiatrists, 8 psychologists, 2 nurse  mental health workers, 3 clinic managers, 1 program evaluator, 4  social workers, 2 psychiatric nurses, 1 administrative assistant. | Canada | Local | General population | General Mental Health | Telehealth | Guided |
| Sinclair et al., (66) | 2013 | Interview | Qualitative | In depth interview | 21 rural clinicians  general practitioners, psychologists,psychiatrists,  clinical social workers | Australia | Local | General population | General Mental Health | Internet of things | Not specified |
| Skime et al., (67) | 2022 | Survey | Mixed methods | Open-ended questions | 40 patients | United States | Local | General population | General Mental Health | Telehealth | Guided |
| Sogomonjan et al., (68) | 2019 | Survey | Qualitative | Semi-structured Interviews | 20 healthcare professionals  18 family physicians  1 psychologist 1 psychiatrist | Estonia | Local | General population | Depression | Computerised CBT | Not specified |
| Steare et al., (69) | 2021 | Interview | Qualitative | Semi-structured Interviews | 21 service users | UK | Local | General population | General Mental Health | Apps | Guided |
| Stjernsward et al., (70) | 2017 | Interview | Qualitative | Not specified | 15 participants of families living with mental health problems  5 parents  5 partners  2 siblings  1 adult child  2 relatives/friends | Sweden | Local | General population | General Mental Health | Web based mindfulness | Not specified |
| Sturk et al., (71) | 2019 | Focus Group | Qualitative | Not specified | 43 practitioners working in mental health set-  tings | Australia | Local | General population | General Mental Health | Web Based program/platform | Not specified |
| Taimen et al.,(72) | 2018 | Survey | Mixed methods | Not specified | 412 physicians | Finland | Local | General population | General Mental Health | Computerised CBT | Not specified |
| Tobbit et al., (73) | 2019 | Survey | Mixed methods | Semi-structured Interviews | 97 service users: 81 patients, 16 staff | UK | Local | General population | General Mental Health | Mobile Health | Not specified |
| Tonnies et al., (74) | 2021 | Interview | Qualitative | Semi-structured Interviews | 15 stakeholders: health insurances, governmental bodies, clinicians’ professional associations, and patient representatives | Germany | Local | General population | General Mental Health | Video consultation | Guided |
| Town et al., (75) | 2017 | Interview | Qualitative | Semi-structured Interviews | 6 employees community child mental health service | UK | Local | Children | General Mental Health | Web Based program/platform | Guided |
| Traube et al., (76) | 2021 | Interview | Qualitative | Semi-structured Interviews | 15 social work students | United States | Local | General population | General Mental Health | Telehealth | Guided |
| Uscher Pines et al., (77) | 2020 | Interview | Qualitative | Semi-structured Interviews | 20 health center leaders | United States | Local | General population | General Mental Health | Telehealth | Guided |
| Volpe et al., (78) | 2014 | Focus Group | Qualitative | Not specified | Focus group with  Nunavut staff (psychiatric nurses,  social workers, child and youth workers, community  wellness workers) | Canada | Local | Nunavut children | General Mental Health | Telehealth | Guided |
| Wozney et al., (79) | 2017 | Interview | Qualitative | Semi-structured Interviews | 31 Key informants:  13 academics  9 organisations  6 national government agencies  3 e-mental health companies | Canada Australia UK Netherlands New Zeland  US | Global | General population | General Mental Health | General Digital Health | Not specified |
| Wynn et al., (80) | 2012 | Interview | Qualitative | Not specified | six clinical psychologists | Norway | Local | General population | General Mental Health | Video consultation | Guided |
| Ye et al., (81) | 2021 | Implementation | Mixed methods | Open-ended questions | 19 Korean Immigrants | United States | Local | Migrants | General Mental Health | Video consultation | Guided |

Table A8: Barriers and facilitators descriptive themes and representative quotes

| **Domain** | **Descriptive theme** | **Barriers** | **Facilitators** |
| --- | --- | --- | --- |
| Cognitive  Behavioural  Attitudinal Emotional | Attitude and Beliefs | “I stopped my treatment because on the internet...” and he then concluded that eHealth “serves to make people sicker.” (45) | “it also makes it a nice tool for people who don't want to talk about mental health among men. It's still a big stigma right now. Not a lot of people that want to admit to it, but it’s nice that it’s there” (16) |
|  | Knowledge, Education and Training | "I feel like it does not come across professionally to set up such an eHealth module with someone if I would not know exactly how it works. I find that unacceptable.” (17) | “Offer continuous training...Create awareness and empowerment of patient and professional before mHealth implementation.” (1) |
| Patient | Gender and Cultural Sensitivity | “Participants also identified areas where they perceived resources to be lacking, specifically for LGBTIQ and CALD consumers, survivors of domestic violence, Indigenous men and young carers” (71) | ‘I felt comfortable and good when I had treatment in Korean language via the teleconference devices.’’ (81) |
|  | Determinants of Health | “Supervisors were largely aware that technology- enabled services would not be acceptable to clinicians and clients across the board, and that specific staff members and client subpopulations who are more technologically-savvy would be more interested in piloting such services” (33) | “The phone itself was a significant perceived benefit (“There is a huge incentive for our clients to have a free phone…” (34) |
|  | Patient choice, preferences, and access | Page 7: “I am most comfortable with books and printed materials. And the Internet, I'm okay with that, I mean I Google and do that, but I find that frustrating too because there are so many options.” (22) | ‘You can go home, it's there. You don't have to drive anywhere. You don't have to book an appointment. It's just available anytime…’" "‘…it (the e- intervention) was easy for me because I sat at my laptop, in my room when I had time. … And the additional modules that you could do, that was nice be- cause, like, that week I could decide…I am going to do this module!’ (20) |
| Professional and interpersonal | Patient-provider relationship, power, and trust | “…then you don’t have the one-on-one contact and that is the power of care . . . If you want to help someone, then you should do that from your heart, then you should do that with love. Otherwise, you cannot help the person. That isn’t the case with eHealth. A computer cannot feel love…that’s how it is with eHealth. You never saw me. It is very clinical. A machine…” (35) | It creates that third party saying, “this is what you said,” so it helps to preserve the therapeutic relationship in terms of not being critical. (14) |
| Guidelines and evidence | Evidence based and implementation | Participants were generally unsure about the evidence base and effectiveness of e-mental health; however, there was optimism about its potential and consensus that more information and research was needed." (52) | Awareness and informed decision-making are necessary to accelerate the implementation of eMH solutions (19) |
|  | Medical safety and crisis management | If I am home at night and I read the message that someone is suicidal, what do I do? So that aggravates matters, because you feel like you always have to be available and you also wonder “where do my responsibilities lie?" (17) | "some participants commented on the concern for a safety plan or the need for structures to be in place should a critical situation arise at the client’s location. (65) |
|  | Technology design, usability, and content | Therapists reported that the ICBT program is highly standardized and not designed for blending with face-to-face care or adapted for patients who need more or less support or time to complete the program. (23) | The use of visual aids to help explain complex mental health concepts and the inclusion of aesthetically engaging content was thought to be particularly important." (52) |
|  | Personalisation and patient-centred care | The Internet-delivered program might be cumbersome to sign in and requires very good commit- ment and self-control skills. People are not used to treating themselves using for that purposes of digital therapies, especially older people. A depressed patient often is demotivated and has difficulties with his/her daily routine. Additional tasks might seem to be as an excessive burden and an additional stressor too (68) | For me personally, I’d rather talk online. You know, if people aren’t going to talk to someone [face-to-face] then with an app they can still deal with their problems. [Participant 9] It’s easier for me to type than speak cos I was brought up with computers." (8) |
| Support and resources | Universal coverage and financial protection | "Findings suggested that unreliable coverage initiates barriers for physicians to refer patients to eMental Health technologies because they do not want the cost to patients to come from out-of-pocket.” (79) | Finally, integration was found to be vital to establish a sustainable funding base for the service through re- imbursement, co-payment or health insurance coverage. (18) |
|  | Innovation, investment and financial risk | “The other question is the updates of these applications, the cost of the updates of these applications, the maintenance, because in the end, while the project is happening it’s ok, but after … you have to see what it would represent in terms of costs.” (10) | “Working with the private sector because they’re way ahead of us in lots of ways and in other ways they’re not because [academics] have the content knowledge, we have the experts but they have the resources and the technology.” (79) |
|  | Time, burden and resources | The most frequently mentioned barrier was a lack of time" "“If it feels like it’s a thing that maybe helps to do something we are already doing better, more efficiently, and then that is going to feel like, “oh great, let’s do this.” But if it feels like an additional thing to do on top of everything we are already doing, that’s hard.” (15) | As another method of improving the inner setting, some participants recommended therapists be rewarded for providing ICBT (e.g., educational opportunities, monetary reward. (23) |
| System and Process | Policy, Regulation, Reforms | "There was some skepticism as to whether the necessary political commitment on the part of both national and international decision makers is substantial enough to ac- tually implement change” (79) | “Participants suggested that effective and ethical e-mental health use should be governed by organizational policies, guidelines, and frameworks.” (52) |
|  | Data protection, security, and privacy | “Privacy is also a topic of high concern within the population and among professionals. As long as there is a perceived uncertainty regarding data storage and protection, acceptance of eMH solutions will most likely remain low” (19) | I think, for me personally, an important aspect of such an app would be its anonymity, that they don't collect any personal data. Because you already share so much personal information in all kinds of pla- ces. And I wouldn't want Facebook to get this [mental health] data of me as well because that's definitely none of their business.” (20) |
|  | Governance, Leadership and Management | “New technologies cannot be implanted unilaterally by the IT team. Health professionals are those who really know what patients need. There has to be an alignment of needs between IT team, patients and health professionals.” (1) | “Finally, the providers noted that access to regular, reﬂective supervision and peer support made them have more comfort when they encountered challenges in working with patients because they knew they had a network with which to troubleshoot issues.” (76) |
|  | Mental Healthcare integration and treatment pathways | “The implementation of the app in private practice is easier...You can charge each visit 2 euros more, for example, and offer patients complementary treatment with the app... In public hospitals this option does not exist at the moment, as there is a lack of budget and of direct interest in mHealth.” (3) | **“**like support/help with, also to make clear boundaries around expectations and commitment, to plan when they will use the programme, and offer telephone reviews weekly or bi-weekly’ (41) |
|  | Public and private mental healthcare systems | Although progress has been made to secure reimbursement for telemental health services from Medicare, Medicaid, and private health plans, respondents still question whether these efforts have gone far enough to provide uniform coverage across third-party payers and states. (30) | “There seems to be such a gap between what’s happening in the academic world and what’s happening in the community, kind of delivery level and also what people are saying they need. If we involved all stakeholders [academics, health providers, end users, private sector] right from the beginning we could probably avoid making a product that we just hope is relevant for someone; I think we forget to add that bit.” (79) |

Table A9: Studies included in each domain (CERQual assessment)

| **Domain** | **Descriptive theme** | **Studies contributing** | **Citation** |
| --- | --- | --- | --- |
| Cognitive  Behavioural  Attitudinal Emotional | Attitude and Beliefs | **n=37** | (1) (3) (8) (11) (13) (15) (16) (18) (20) (23) (24) (25) (29) (31) (34) (35) (37) (38) (40) (45) (47) (49) (52) (53) (55) (56) (57) (59) (61) (63) (67) (68) (71) (72) (75) (77) (80) |
|  | Knowledge, Education and Training | **n=48** | (1) (2) (3) (6) (7) (10) (12) (13) (14) (15) (17) (19) (23) (25) (27) (36) (31) (34) (35) (39) (40) (41) (43) (44) (46) (47) (48) (49) (50) (52) (53) (55) (54) (56) (57) (58) (60) (61) (63) (64) (67) (72) (73) (76) (78) (79) (80) (81) |
| Patient | Gender and Cultural sensitivity | **n=10** | (9) (11) (14) (16) (19) (46) (49) (52) (55) (71) |
|  | Determinants of Health | **n=26** | (1) (3) (4) (9) (10) (15) (17) (22) (30) (31) (33) (34) (36) (38) (41) (43) (47) (52) (53) (55) (59) (65) (66) (69) (71) (73) |
|  | Patient choice, preferences and access | **n=34** | (1) (4) (6) (7) (8) (9) (13) (15) (16) (20) (22) (24) (31) (33) (35) (36) (37) (39) (43) (44) (45) (46) (55) (57) (60) (61) (63) (66) (67) (68) (70) (76) (79) (81) |
| Professional and interpersonal | Patient-provider relationship | **n=44** | (1) (3) (4) (5) (6) (7) (8) (13) (17) (18) (19) (20) (23) (24) (25) (29) (32) (33) (34) (35) (39) (41) (43) (44) (26) (45) (47) (48) (49) (50) (51) (52) (53) (59, 61) (63) (66) (67) (68) (69) (75) (77) (79) (80) |
|  | Trust | **n=30** | (1) (2) (4) (5) (13) (14) (23) (24) (31) (37) (38) (39) (40) (43) (44) (45) (52) (53) (56) (58) (62) (61) (63) (65) (66) (69) (75) (79) (80) (81) |
|  | Power | **n=19** | (4) (5) (7) (8) (9) (14) (15) (31) (34) (35) (39) (45) (47) (52) (58) (66) (71) (75) (79) |
| Guidelines and evidence | Evidence based and implementation | **n=17** | (1) (2) (4) (5) (12) (14) (19) (23) (33) (41) (47) (50) (52) (62) (68) (72) (79) |
|  | Medical safety and crisis management | **n=16** | (37) (4) (5) (17) (33) (34) (40) (43) (44) (45) (58) (59) (64) (65) (66) (72) |
|  | Technology design, usability and content | **n=30** | (1) (2) (4) (5) (9) (11) (12) (14) (15) (17) (19) (22) (23) (29) (34) (39) (40) (42) (43) (49) (52) (55) (56) (58) (59) (68) (69) (71) (75) (81) |
|  | Personalisation and patient-centred care | **n=38** | (3) (4) (7) (8) (9) (10) (11) (17) (18) (20) (22) (21) (23) (29) (31) (33) (38) (41) (42) (44) (45) (47) (49) (51) (52) (55) (58) (61) (63) (65) (66) (68) (69) (72) (74) (75) (77) |
| Support and resources | Universal coverage and financial protection | **n=22** | (2) (5) (7) (10) (18) (19) (25) (27) (30) (31) (32) (33) (34) (36) (38) (26) (46) (52) (58) (76) (77) (79) |
|  | Innovation, investment and financial risk | **n=13** | (2) (10) (12) (19) (23) (25) (27) (30) (34) (26) (47) (62) (79) |
|  | Time, burden and resources | **n=46** | (1) (2) (3) (5) (10) (13) (14) (15) (17) (18) (20) (22) (23) (25) (27) (29) (29) (30) (31) (34) (36) (40) (43) (41) (26) (46) (47) (48) (49) (50) (52) (53) (54) (56) (57) (58) (60) (62) (61) (63) (68) (69) (74) (75) (78) (79) (80) |
| System and Process | Policy, Regulation, Reforms | **n=16** | (1) (18) (19) (25) (27) (28) (30) (33) (34) (45) (48) (52) (55) (72) (74) (79) |
|  | Data protection, security, and privacy | **n=34** | (1) (4) (5) (8) (9) (13) (15) (19) (20) (22) (24) (33) (34) (37) (41) (42) (43) (44) (47) (49) (50) (52) (53) (60) (64) (68) (69) (71) (72) (74) (77) (78) (80) (81) |
|  | Governance, Leadership and Management | **n=25** | (1) (2) (3) (14) (15) (18) (23) (25) (27) (29) (31) (34) (35) (26) (46) (47) (49) (52) (54) (62) (66) (74) (75) (76) (79) |
|  | Mental Healthcare integration and treatment pathways | **n=30** | (2) (4) (7) (11) (14) (15) (16) (18) (23) (24) (29) (30) (31) (38) (39) (41) (44) (26) (48) (49) (52) (54) (56) (59) (69) (71) (74) (75) (79) |
|  | Public and private mental healthcare systems | **n=4** | (79) (30) (1) (3) |

Table A10: Grade CERQual assessment of confidence in the evidence

| **Descriptive theme** | **Methodological limitation** | **Coherence** | **Adequacy** | **Relevance** | **CERQual assessment of confidence in the evidence** |
| --- | --- | --- | --- | --- | --- |
| Attitude and Beliefs | No or very minor concerns | Moderate concerns | Minor concerns | No or very minor concerns | Minor concerns |
| Knowledge, Education and Training | No or very minor concerns | Moderate concerns | Minor concerns | No or very minor concerns | Minor concerns |
| Gender and Cultural Sensitivity | No or very minor concerns | No or very minor concerns | No or very minor concerns | No or very minor concerns | No or very minor concerns |
| Determinants of Health | No or very minor concerns | Minor concerns | No or very minor concerns | No or very minor concerns | No or very minor concerns |
| Patient choice, preferences and access | No or very minor concerns | Minor concerns | No or very minor concerns | No or very minor concerns | No or very minor concerns |
| Patient-provider relationship | No or very minor concerns | Minor concerns | No or very minor concerns | No or very minor concerns | No or very minor concerns |
| Trust | No or very minor concerns | Minor concerns | No or very minor concerns | No or very minor concerns | No or very minor concerns |
| Power | No or very minor concerns | Minor concerns | No or very minor concerns | No or very minor concerns | No or very minor concerns |
| Evidence based and implementation | No or very minor concerns | No or very minor concerns | No or very minor concerns | No or very minor concerns | No or very minor concerns |
| Medical safety and crisis management | Minor concerns | Minor concerns | No or very minor concerns | Minor concerns | Minor concerns |
| Technology design, usability and content | No or very minor concerns | Minor concerns | No or very minor concerns | No or very minor concerns | No or very minor concerns |
| Personalisation and patient-centered care | No or very minor concerns | Minor concerns | No or very minor concerns | No or very minor concerns | No or very minor concerns |
| Universal coverage and financial protection | No or very minor concerns | Minor concerns | Minor concerns | No or very minor concerns | Minor concerns |
| Innovation, investment and financial risk | No or very minor concerns | No or very minor concerns | Minor concerns | No or very minor concerns | No or very minor concerns |
| Time, burden and resources | No or very minor concerns | Minor concerns | No or very minor concerns | No or very minor concerns | No or very minor concerns |
| Policy, Regulation, Reforms | No or very minor concerns | No or very minor concerns | Minor concerns | No or very minor concerns | No or very minor concerns |
| Data protection, security and privacy | No or very minor concerns | Minor concerns | No or very minor concerns | No or very minor concerns | No or very minor concerns |
| Governance, Leadership and Management | No or very minor concerns | No or very minor concerns | No or very minor concerns | No or very minor concerns | No or very minor concerns |
| Mental Healthcare integration and treatment pathways | No or very minor concerns | Minor concerns | No or very minor concerns | No or very minor concerns | No or very minor concerns |
| Public and private mental healthcare systems | No or very minor concerns | Minor concerns | No or very minor concerns | No or very minor concerns | No or very minor concerns |
| **Criteria to assess:**  **Methodological limitation** = Clarity of described method (yes, no; Table 8), Research influence (medium, low; Table 6), Participants voice represented (medium, low; Table 6), Interpretation of results (medium, low; Table 6);  **Coherence** = Theoretical framework (medium, low; Table 6);  **Adequacy** = Sample <15 (Table 8)  **Relevance**= Relevant to one of more levels and domains of the mental health system (Figure 1,2).  The classification has been defined using the following weights for each domain: **Methodological limitation** =0.4; **Coherence** = 0.2; **Adequacy** =0.2; R**elevance**= 0.2 | | | | | |
| **Classification**: | | | | | |
| **No or very minor concerns**: Method= No or very minor concern and max 1 minor concern on other domains | | | | | |
| **Minor concerns**: Method= No or very minor concern and max 1 moderate concern on other domains OR Method= minor concern and max 2 minor concern on other domains | | | | | |
| **Moderate concerns**: Method= No or very minor concern and > 1 moderate concern on other domains OR Method=minor or moderate concern and 4 moderate concern on other domains | | | | | |
| **Serious concerns**: Method= moderate concern and >= 1 serious concern on other domains | | | | | |

**Appendix References**

1. Anastasiadou D, Folkvord F, Serrano-Troncoso E, Lupiañez-Villanueva F. Mobile Health Adoption in Mental Health: User Experience of a Mobile Health App for Patients With an Eating Disorder. JMIR Mhealth Uhealth. 2019;7(6):e12920.

2. Bauer MS, Krawczyk L, Tuozzo K, Frigand C, Holmes S, Miller CJ, et al. Implementing and Sustaining Team-Based Telecare for Bipolar Disorder: Lessons Learned from a Model-Guided, Mixed Methods Analysis. Telemed J E Health. 2018;24(1):45-53.

3. Bennett-Levy J, Singer J, DuBois S, Hyde K. Translating E-Mental Health Into Practice: What Are the Barriers and Enablers to E-Mental Health Implementation by Aboriginal and Torres Strait Islander Health Professionals? J Med Internet Res. 2017;19(1):e1.

4. Berry N, Bucci S, Lobban F. Use of the Internet and Mobile Phones for Self-Management of Severe Mental Health Problems: Qualitative Study of Staff Views. JMIR Ment Health. 2017;4(4):e52.

5. Blease C, Locher C, Leon-Carlyle M, Doraiswamy M. Artificial intelligence and the future of psychiatry: Qualitative findings from a global physician survey. Digit Health. 2020;6:2055207620968355.

6. Bleyel C, Hoffmann M, Wensing M, Hartmann M, Friederich HC, Haun MW. Patients' Perspective on Mental Health Specialist Video Consultations in Primary Care: Qualitative Preimplementation Study of Anticipated Benefits and Barriers. J Med Internet Res. 2020;22(4):e17330.

7. Bruno R, Abbott J-AM. Australian health professionals’ attitudes toward and frequency of use of internet supported psychological interventions. International Journal of Mental Health. 2015;44(1-2):107-23.

8. Bucci S, Morris R, Berry K, Berry N, Haddock G, Barrowclough C, et al. Early Psychosis Service User Views on Digital Technology: Qualitative Analysis. JMIR Ment Health. 2018;5(4):e10091.

9. Burchert S, Alkneme MS, Bird M, Carswell K, Cuijpers P, Hansen P, et al. User-Centered App Adaptation of a Low-Intensity E-Mental Health Intervention for Syrian Refugees. Front Psychiatry. 2018;9:663.

10. Cárdenas P, Bartels SM, Cruz V, Gáfaro L, Uribe-Restrepo JM, Torrey WC, et al. Perspectives, Experiences, and Practices in the Use of Digital Information Technologies in the Management of Depression and Alcohol Use Disorder in Health Care Systems in Colombia. Qual Health Res. 2020;30(6):906-16.

11. Cheng VWS, Piper SE, Ottavio A, Davenport TA, Hickie IB. Recommendations for Designing Health Information Technologies for Mental Health Drawn From Self-Determination Theory and Co-design With Culturally Diverse Populations: Template Analysis. J Med Internet Res. 2021;23(2):e23502.

12. Clarke A, Adamson J, Sheard L, Cairns P, Watt I, Wright J. Implementing electronic patient record systems (EPRs) into England's acute, mental health and community care trusts: a mixed methods study. BMC Med Inform Decis Mak. 2015;15:85.

13. Dederichs M, Weber J, Pischke CR, Angerer P, Apolinario-Hagen J. Exploring medical students' views on digital mental health interventions: A qualitative study. Internet Interventions. 2021;25:100398.

14. Dingwall KM, Puszka S, Sweet M, Nagel T. “Like Drawing Into Sand”: Acceptability, Feasibility, and Appropriateness of a New e‐Mental Health Resource for Service Providers Working With A boriginal and T orres S trait I slander People. Australian Psychologist. 2015;50(1):60-9.

15. Dinkel D, Caspari JH, Fok L, Notice M, Johnson DJ, Watanabe-Galloway S, et al. A qualitative exploration of the feasibility of incorporating depression apps into integrated primary care clinics. Translational Behavioral Medicine. 2021;11(9):1708-16.

16. Eccles H, Nannarone M, Lashewicz B, Attridge M, Marchand A, Aiken A, et al. Perceived Effectiveness and Motivations for the Use of Web-Based Mental Health Programs: Qualitative Study. J Med Internet Res. 2020;22(7):e16961.

17. Feijt MA, de Kort YA, Bongers IM, WA IJ. Perceived Drivers and Barriers to the Adoption of eMental Health by Psychologists: The Construction of the Levels of Adoption of eMental Health Model. J Med Internet Res. 2018;20(4):e153.

18. Folker AP, Mathiasen K, Lauridsen SM, Stenderup E, Dozeman E, Folker MP. Implementing internet-delivered cognitive behavior therapy for common mental health disorders: A comparative case study of implementation challenges perceived by therapists and managers in five European internet services. Internet Interv. 2018;11:60-70.

19. Gaebel W, Lukies R, Kerst A, Stricker J, Zielasek J, Diekmann S, et al. Upscaling e-mental health in Europe: a six-country qualitative analysis and policy recommendations from the eMEN project. Eur Arch Psychiatry Clin Neurosci. 2021;271(6):1005-16.

20. Gericke F, Ebert DD, Breet E, Auerbach RP, Bantjes J. A qualitative study of university students' experience of internet-based CBT for depression. [References]. Counselling & Psychotherapy Research. 2021;21(4):792-804.

21. Gordon D, Hensel J, Bouck Z, Desveaux L, Soobiah C, Saragosa M, et al. Developing an explanatory theoretical model for engagement with a web-based mental health platform: Results of a mixed methods study. [References]. BMC Psychiatry. 2021;21:417.

22. Gould CE, Loup J, Kuhn E, Beaudreau SA, Ma F, Goldstein MK, et al. Technology use and preferences for mental health self-management interventions among older veterans. Int J Geriatr Psychiatry. 2020;35(3):321-30.

23. Hadjistavropoulos HD, Nugent MM, Dirkse D, Pugh N. Implementation of internet-delivered cognitive behavior therapy within community mental health clinics: a process evaluation using the consolidated framework for implementation research. BMC Psychiatry. 2017;17(1):331.

24. Hawke LD, Sheikhan NY, MacCon K, Henderson J. Going virtual: youth attitudes toward and experiences of virtual mental health and substance use services during the COVID-19 pandemic. BMC Health Services Research. 2021;21(1):340.

25. Hermes EDA, Burrone L, Heapy A, Martino S, Perez E, Rosenheck R, et al. Beliefs and Attitudes About the Dissemination and Implementation of Internet-Based Self-Care Programs in a Large Integrated Healthcare System. Adm Policy Ment Health. 2019;46(3):311-20.

26. Howland M, Tennant M, Bowen DJ, Bauer AM, Fortney JC, Pyne JM, et al. Psychiatrist and Psychologist Experiences with Telehealth and Remote Collaborative Care in Primary Care: A Qualitative Study. J Rural Health. 2021;37(4):780-7.

27. Jonk YC, Burgess A, Williamson ME, Thayer D, MacKenzie J, McGuire C, et al. Telehealth Use in a Rural State: A Mixed-Methods Study Using Maine's All-Payer Claims Database. J Rural Health. 2021;37(4):769-79.

28. Kenicer D, McClay CA, Williams C. A national survey of health service infrastructure and policy impacts on access to computerised CBT in Scotland. BMC Med Inform Decis Mak. 2012;12:102.

29. Kurki M, Anttila M, Koivunen M, Marttunen M, Välimäki M. Nurses' experiences of the use of an Internet-based support system for adolescents with depressive disorders. Inform Health Soc Care. 2018;43(3):234-47.

30. Lambert D, Gale J, Hartley D, Croll Z, Hansen A. Understanding the Business Case for Telemental Health in Rural Communities. J Behav Health Serv Res. 2016;43(3):366-79.

31. LaMonica HM, Milton A, Braunstein K, Rowe SC, Ottavio A, Jackson T, et al. Technology-Enabled Solutions for Australian Mental Health Services Reform: Impact Evaluation. JMIR Form Res. 2020;4(11):e18759.

32. Landes SJ, Pitcock JA, Harned MS, Connolly SL, Meyers LL, Oliver CM. Provider perspectives on delivering dialectical behavior therapy via telehealth during COVID-19 in the Department of Veterans Affairs. [References]. Psychological Services. 2021(Pagination).

33. Lattie EG, Nicholas J, Knapp AA, Skerl JJ, Kaiser SM, Mohr DC. Opportunities for and Tensions Surrounding the Use of Technology-Enabled Mental Health Services in Community Mental Health Care. Adm Policy Ment Health. 2020;47(1):138-49.

34. Lord S, Moore SK, Ramsey A, Dinauer S, Johnson K. Implementation of a Substance Use Recovery Support Mobile Phone App in Community Settings: Qualitative Study of Clinician and Staff Perspectives of Facilitators and Barriers. JMIR Ment Health. 2016;3(2):e24.

35. Lorenz-Artz K, Bierbooms J, Bongers I. Integrating eHealth within a Transforming Mental Healthcare Setting: A Qualitative Study into Values, Challenges, and Prerequisites. Int J Environ Res Public Health. 2021;18(19):29.

36. Magal T, Negev M, Kaphzan H. Attitudinal Barriers Hindering Adoption of Telepsychiatry among Mental Healthcare Professionals: Israel as a Case-Study. Int J Environ Res Public Health. 2021;18(23):28.

37. Mar MY, Neilson EK, Torchalla I, Werker GR, Laing A, Krausz M. Exploring e-mental health preferences of generation Y. Journal of Technology in Human Services. 2014;32(4):312-27.

38. Margolis K, Kelsay K, Talmi A, McMillan H, Fraley MC, Thomas JFF. A multidisciplinary, team-based teleconsultation approach to enhance child mental health services in rural pediatrics. Journal of Educational and Psychological Consultation. 2018;28(3):342-67.

39. Matanov A, McNamee P, Akther S, Barber N, Bird V. Acceptability of a technology-supported and solution-focused intervention (DIALOG+) for chronic depression: Views of service users and clinicians. [References]. BMC Psychiatry. 2021;21:263.

40. Matthews EB. Integrating the Electronic Health Record into Behavioral Health Encounters: Strategies, Barriers, and Implications for Practice. Adm Policy Ment Health. 2017;44(4):512-23.

41. Meisel SF, Drury H, Perera-Delcourt RP. Therapists’ attitudes to offering eCBT in an inner-city IAPT service: a survey study. The Cognitive Behaviour Therapist. 2018;11.

42. Melcher J, Camacho E, Lagan S, Torous J. College student engagement with mental health apps: analysis of barriers to sustained use. J Am Coll Health. 2020:1-7.

43. Melia R, Monahan L, Duggan J, Bogue J, O'Sullivan M, Young K, et al. Exploring the experiences of mental health professionals engaged in the adoption of mobile health technology in Irish mental health services. BMC Psychiatry. 2021;21(1):412.

44. Mercado M, Little V. Clinicians' perceptions of telephone-delivered mental health services. [References]. The Journal of Mental Health Training, Education and Practice. 2020;15(2):104-13.

45. Morgiève M, Sebbane D, De Rosario B, Demassiet V, Kabbaj S, Briffault X, et al. Analysis of the Recomposition of Norms and Representations in the Field of Psychiatry and Mental Health in the Age of Electronic Mental Health: Qualitative Study. JMIR Ment Health. 2019;6(10):e11665.

46. Newman L, Bidargaddi N, Schrader G. Service providers' experiences of using a telehealth network 12 months after digitisation of a large Australian rural mental health service. Int J Med Inform. 2016;94:8-20.

47. Orlowski S, Lawn S, Matthews B, Venning A, Wyld K, Jones G, et al. The promise and the reality: a mental health workforce perspective on technology-enhanced youth mental health service delivery. BMC Health Serv Res. 2016;16(1):562.

48. Perry K, Gold S, Shearer EM. Identifying and addressing mental health providers' perceived barriers to clinical video telehealth utilization. J Clin Psychol. 2020;76(6):1125-34.

49. Pithara C, Farr M, Sullivan SA, Edwards HB, Hall W, Gadd C, et al. Implementing a Digital Tool to Support Shared Care Planning in Community-Based Mental Health Services: Qualitative Evaluation. J Med Internet Res. 2020;22(3):e14868.

50. Pokhrel P, Karmacharya R, Taylor Salisbury T, Carswell K, Kohrt BA, Jordans MJD, et al. Perception of healthcare workers on mobile app-based clinical guideline for the detection and treatment of mental health problems in primary care: a qualitative study in Nepal. BMC Med Inform Decis Mak. 2021;21(1):21.

51. Pung A, Fletcher SL, Gunn JM. Mobile app use by primary care patients to manage their depressive symptoms: qualitative study. Journal of medical Internet research. 2018;20(9):e10035.

52. Puszka S, Dingwall KM, Sweet M, Nagel T. E-mental health innovations for Aboriginal and Torres Strait Islander Australians: a qualitative study of implementation needs in health services. JMIR mental health. 2016;3(3):e5837.

53. Pywell J, Vijaykumar S, Dodd A, Coventry L. Barriers to older adults’ uptake of mobile-based mental health interventions. Digital health. 2020;6:2055207620905422.

54. Raphiphatthana B, Sweet M, Puszka S, Dingwall K, Nagel T. Evaluation of a three-phase implementation program in enhancing e-mental health adoption within Indigenous primary healthcare organisations. BMC health services research. 2020;20(1):1-16.

55. Raphiphatthana B, Sweet M, Puszka S, Whitty M, Dingwall K, Nagel T. Evaluation of electronic mental health implementation in Northern Territory services using the integrated “Promoting Action on Research Implementation in Health Services” framework: Qualitative study. JMIR Mental Health. 2020;7(5):e14835.

56. Reger GM, Browne KC, Campellone TR, Simons C, Kuhn E, Fortney JC, et al. Barriers and facilitators to mobile application use during PTSD treatment: Clinician adoption of PE coach. Professional Psychology: Research and Practice. 2017;48(6):510.

57. Renfrew ME, Morton DP, Northcote M, Morton JK, Hinze JS, Przybylko G. Participant perceptions of facilitators and barriers to adherence in a digital mental health intervention for a nonclinical cohort: Content analysis. [References]. Journal of Medical Internet Research. 2021;23(4).

58. Richards P, Simpson S, Bastiampillai T, Pietrabissa G, Castelnuovo G. The impact of technology on therapeutic alliance and engagement in psychotherapy: The therapist's perspective. Clinical Psychologist. 2018;22(2):171-81.

59. Rodda S, Merkouris S, Lavis T, Smith D, Lubman D, Austin D, et al. The therapist experience of internet delivered CBT for problem gambling: Service integration considerations. Internet interventions. 2019;18:100264.

60. Rozental A, Kothari R, Wade T, Egan S, Andersson G, Carlbring P, et al. Reconsidering perfect: a qualitative study of the experiences of internet-based cognitive behaviour therapy for perfectionism. Behav Cogn Psychother. 2020;48(4):432-41.

61. Rushton K, Ardern K, Hopkin E, Welsh C, Gellatly J, Faija C, et al. 'I didn't know what to expect': Exploring patient perspectives to identify targets for change to improve telephone-delivered psychological interventions. BMC Psychiatry. 2020;20(1):156.

62. Rushton K, Fraser C, Gellatly J, Brooks H, Bower P, Armitage CJ, et al. A case of misalignment: the perspectives of local and national decision-makers on the implementation of psychological treatment by telephone in the improving access to psychological therapies service. BMC health services research. 2019;19(1):1-12.

63. Schneider J, Sarrami Foroushani P, Grime P, Thornicroft G. Acceptability of online self-help to people with depression: users' views of MoodGYM versus informational websites. J Med Internet Res. 2014;16(3):e90.

64. Shealy KM, Davidson TM, Jones AM, Lopez CM, de Arellano MA. Delivering an evidence-based mental health treatment to underserved populations using telemedicine: The case of a trauma-affected adolescent in a rural setting. Cognitive and Behavioral Practice. 2015;22(3):331-44.

65. Simms DC, Gibson K, O'Donnell S. To use or not to use: Clinicians' perceptions of telemental health. Canadian Psychology/Psychologie canadienne. 2011;52(1):41.

66. Sinclair C, Holloway K, Riley G, Auret K. Online mental health resources in rural Australia: clinician perceptions of acceptability. J Med Internet Res. 2013;15(9):e193.

67. Skime MK, Puspitasari AJ, Gentry MT, Heredia D, Jr., Sawchuk CN, Moore WR, et al. Patient Satisfaction and Recommendations for Delivering a Group-Based Intensive Outpatient Program via Telemental Health during the COVID-19 Pandemic. JMIR Mental Health. 2021;02:02.

68. Sogomonjan M, Kerikmäe T, Ööpik P, Ross P. A report on the survey. Attitudes of Estonian healthcare professionals to internet-delivered cognitive behavioural therapy. Cogent Psychology. 2019;6(1):1637623.

69. Steare T, Giorgalli M, Free K, Harju-Seppanen J, Akther S, Eskinazi M, et al. A qualitative study of stakeholder views on the use of a digital app for supported self-management in early intervention services for psychosis. [References]. BMC Psychiatry. 2021;21:311.

70. Stjernswärd S, Hansson L. User value and usability of a web-based mindfulness intervention for families living with mental health problems. Health Soc Care Community. 2017;25(2):700-9.

71. Sturk H, Crowther R, Kavanagh DJ. Head to health: Practitioner perceptions of the new digital mental health gateway. Aust J Rural Health. 2019;27(5):448-53.

72. Taiminen HSM, Saraniemi S, Parkinson J. Incorporating digital self-services into integrated mental health care: a physician’s perspective. European Journal of Marketing. 2018.

73. Tobitt S, Percival R. Switched on or switched off? A survey of mobile, computer and Internet use in a community mental health rehabilitation sample. Journal of Mental Health. 2019;28(1):4-10.

74. Tonnies J, Oeljeklaus L, Wensing M, Hartmann M, Friederich HC, Haun MW. Health policy experts' perspectives on implementing mental health specialist video consultations in routine primary care - a qualitative interview study. BMC Health Services Research. 2021;21(1):713.

75. Town R, Midgley N, Ellis L, Tempest R, Wolpert M. A qualitative investigation of staff's practical, personal and philosophical barriers to the implementation of a web‐based platform in a child mental health setting. Counselling and Psychotherapy Research. 2017;17(3):218-26.

76. Traube DE, Cederbaum JA, Taylor A, Naish L, Rau A. Telehealth training and provider experience of delivering behavioral health services. The Journal of Behavioral Health Services & Research. 2021;48(1):93-102.

77. Uscher-Pines L, Raja P, Qureshi N, Huskamp HA, Busch AB, Mehrotra A. Use of Tele-Mental Health in Conjunction With In-Person Care: A Qualitative Exploration of Implementation Models. Psychiatr Serv. 2020;71(5):419-26.

78. Volpe T, Boydell KM, Pignatiello A. Mental health services for Nunavut children and youth: evaluating a telepsychiatry pilot project. Rural Remote Health. 2014;14(2):2673.

79. Wozney L, Newton AS, Gehring ND, Bennett K, Huguet A, Hartling L, et al. Implementation of eMental Health care: viewpoints from key informants from organizations and agencies with eHealth mandates. BMC Med Inform Decis Mak. 2017;17(1):78.

80. Wynn R, Bergvik S, Pettersen G, Fossum S. Clinicians' experiences with videoconferencing in psychiatry. Stud Health Technol Inform. 2012;180:1218-20.

81. Ye J, Shim R, Lukaszewski T, Yun K, Kim SH, Ruth G. Telepsychiatry services for Korean immigrants. Telemed J E Health. 2012;18(10):797-802.

82. Richards D, Richardson T. Computer-based psychological treatments for depression: a systematic review and meta-analysis. Clin Psychol Rev. 2012;32(4):329-42.
